# Polyphenols as Antitumor Agents Targeting Key Players in Cancer-Driving Signaling Pathways

**DOI:** 10.3389/fphar.2021.710304

**Published:** 2021-10-20

**Authors:** Manuel Humberto Cháirez-Ramírez, Karen Griselda de la Cruz-López, Alejandro García-Carrancá

**Affiliations:** ^1^ Unidad de Investigación Biomédica en Cáncer, Instituto de Investigaciones Biomédicas, Universidad Nacional Autónoma de México and Instituto Nacional de Cancerología, Secretaría de Salud, Mexico City, Mexico; ^2^ Programa de Doctorado en Ciencias Biomédicas, Instituto de Investigaciones Biomédicas, Universidad Nacional Autónoma de México, Mexico City, Mexico

**Keywords:** polyphenols, signaling pathways, cancer, MAPK, PI3K-AKT pathway, p53, RAS

## Abstract

Polyphenols constitute an important group of natural products that are traditionally associated with a wide range of bioactivities. These are usually found in low concentrations in natural products and are now available in nutraceuticals or dietary supplements. A group of polyphenols that include apigenin, quercetin, curcumin, resveratrol, EGCG, and kaempferol have been shown to regulate signaling pathways that are central for cancer development, progression, and metastasis. Here, we describe novel mechanistic insights on the effect of this group of polyphenols on key elements of the signaling pathways impacting cancer. We describe the protein modifications induced by these polyphenols and their effect on the central elements of several signaling pathways including PI3K, Akt, mTOR, RAS, and MAPK and particularly those affecting the tumor suppressor p53 protein. Modifications of p53 induced by these polyphenols regulate p53 gene expression and protein levels and posttranslational modifications such as phosphorylation, acetylation, and ubiquitination that influence stability, subcellular location, activation of new transcriptional targets, and the role of p53 in response to DNA damage, apoptosis control, cell- cycle regulation, senescence, and cell fate. Thus, deep understanding of the effects that polyphenols have on these key players in cancer-driving signaling pathways will certainly lead to better designed targeted therapies, with less toxicity for cancer treatment. The scope of this review centers on the regulation of key elements of cancer signaling pathways by the most studied polyphenols and highlights the importance of a profound understanding of these regulations in order to improve cancer treatment and control with natural products.

## Introduction

Cancer represents the second cause of death attributable to noncommunicable diseases, after only cardiovascular diseases. Despite the fact that the cancer death rate has been reduced in the last 30 years by about 31%, related to the fact that healthier lifestyle habits improve health status, it continues to be a major concern for public health systems worldwide (Sung et al., 2021). At present, there are numerous treatments for cancer, including surgery, chemotherapy, hormonal therapy, radiation, immune therapy, targeted treatments, nanotechnology, and RNA therapeutics (microRNA and RNAi). Chemotherapeutics have been predominant for systemic cancer treatment; the majority of these are acting to cause DNA damage in order to kill or to inhibit cells from an accelerated rate of division. Chemotherapeutics are administered as single doses or short therapies at the maximal tolerable dose, followed by a treatment-free time that must be observed to allow for the recovery of normal cells (Nurgali et al., 2018). Despite the benefits of chemotherapy, it gives rise to adverse effects including hematological toxicity, alterations of gastrointestinal activity, alopecia, alterations of neurological activity, anaphylaxis, hepatotoxicity, and nephrotoxicity. The adverse effects of systemic chemotherapy are often severe and reduce the quality of life of patients. Although many adverse effects can be prevented with adequate prophylaxis, the toxicity of some agents cannot be controlled; therefore, a dose reduction becomes the only alternative. In this regard, plant-derived natural compounds such as polyphenols may arise as ideal alternatives for single or concomitant therapies for cancer treatment with more effectiveness, safety, and less toxicity.

Plant-derived natural compounds have been used for the prevention and treatment of many diseases. Plants produce a wide range of secondary metabolites that confer on them great adaptability to act as antimicrobial agents, as growth enhancers, in resistance to water stress, as sun screeners, and as an aid to repel predators (Weng et al., 2012). Secondary metabolites include polyphenols with nearly 10,000 known members, composed of several aromatic rings and multiple hydroxyl groups in their structure, with moderate water solubility and considerable antioxidant capacity (Brglez Mojzer et al., 2016). Individuals obtain approximately 1 g/day of polyphenols from their diet; however, this varies according to socioeconomic factors, gender, and the region of the world where people live. More than 800 polyphenols have been identified in food sources, including cereals, cocoa, coffee, tea, wine, and berries (Pérez-Jiménez et al., 2010). Despite the advances in drug discovery and development during the last decades, herbal medicine continues to be used as primary therapy in many developing countries (nearly 4 billion persons) (Ekor, 2014). Regular consumption of polyphenols has been related to beneficial health effects, including regulation of the intestinal microbiota and antiaging effects (Shimizu et al., 2019), a risk reduction of atherosclerosis (Nie et al., 2019), a decrease in the risk of colorectal cancer development (Bahrami et al., 2019), and the modulation of antioxidant enzymes through Nrf2 regulation (Lee et al., 2018). One of the major challenges for the therapeutic use of polyphenols is their low oral bioavailability. The absorption, transportation, bioavailability, and bioactivity of polyphenols are of interest in terms of their use and as new drug candidates. After oral administration, polyphenols pass through the gastrointestinal tract (GI) with absorption in the stomach and small intestine, and some are biotransformed by gut microbiota or by those absorbed during the early stages of digestion by hepatic phase I/II metabolism, prior to reaching the systemic circulation, which may affect bioavailability and bioactivity. Results of importance consider all of these processes and how they will affect the pharmacokinetics and pharmacodynamics of polyphenols. However, accessibility, economic importance, beneficial health effects, and the safety of polyphenols compared to synthetic drugs (Karimi et al., 2015) make them perfect candidates to explore possible therapeutic effects for preventing or treating different types of cancer due to the capacity of polyphenols to modulate multiple signaling pathways such as MAPK and PI3K/Akt and the key proteins involved in cancer development, such as p53 and RAS, rendering a promising expectation regarding these compounds. The present review aimed at focusing on the chemistry, bioavailability, and bioactivities of polyphenols in the key elements involved in cancer development and progression.

## Polyphenols: Their Chemistry and Their Importance in Human Health

### Relevant Members of the Polyphenol Family

**FIGURE 1:**
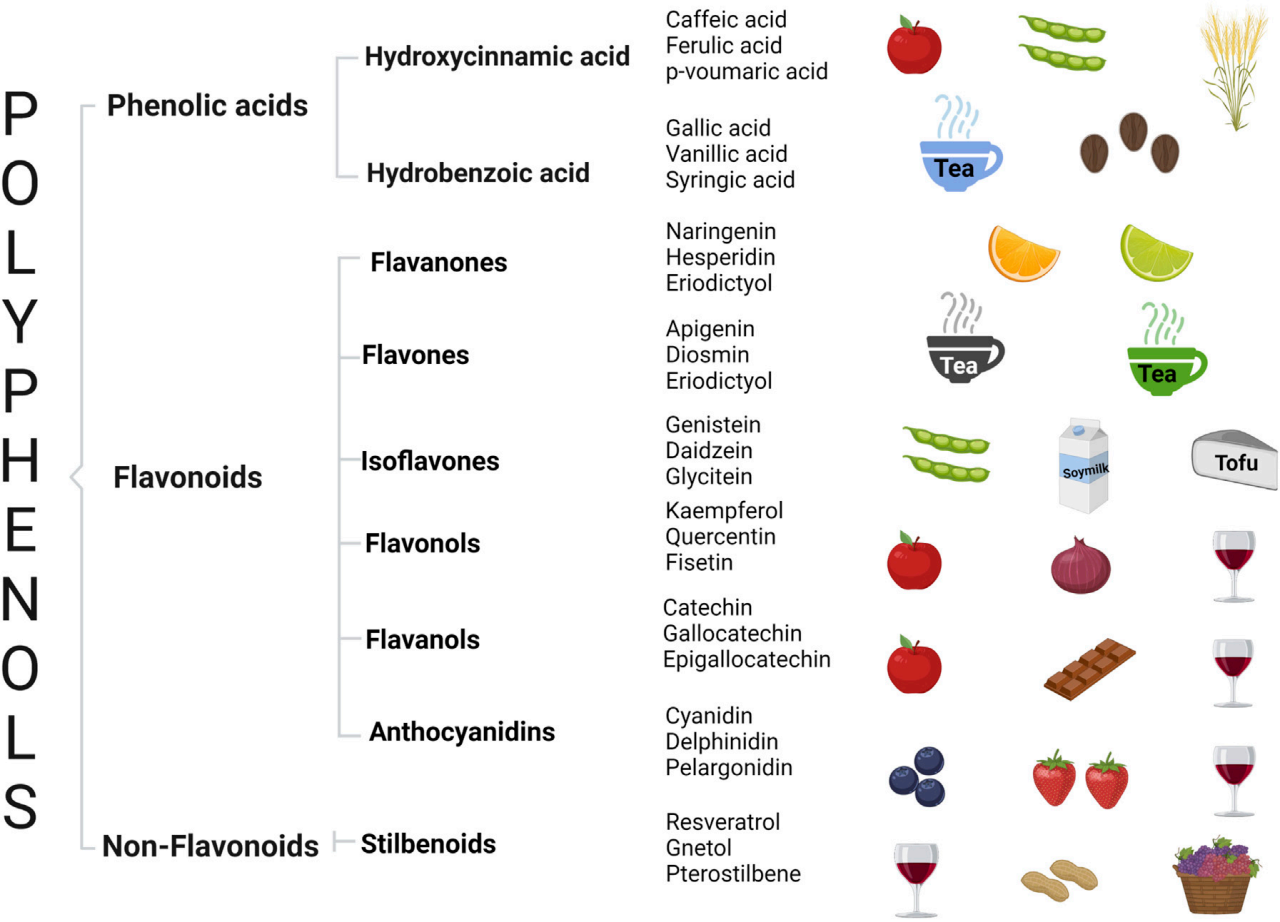
General classification of polyphenols, representative compounds by group, and their associated rich food sources.

Polyphenols are classified as derivatives of shikimic acid/phenylpropanoids (derived from tyrosine and phenylalanine) and polyketide (lacking functional groups related to nitrogen) pathways. For shikimic acid derivatives, phenylpropanoid units serve as the basis for multiple types of polyphenols, such as cinnamic (C6–C3), benzoic acids (C6–C1), flavonoids (C6–C3–C6), proanthocyanidins [(C6–C3–C6)n], stilbenoids (C6–C2–C6), and lignins [(C6–C3)n] (Figure 1) (Pereira et al., 2009; Cirkovic-Velickovic and Stanic-Vucinic, 2018).

#### Phenolic Acids

Phenolic acids are the simplest phenolic compounds, formed of only one phenolic ring with multiple hydroxy or methoxy groups attached to their backbone. Hydroxycinnamic acids are aromatic carboxylic acids with unsaturation in the side chain (commonly of *trans*-configuration) and are more abundant than hydroxybenzoic acids. Cinnamic acids work as phytohormones, which are important components of lignin and the precursors of chalcones, flavonoids, anthocyanins, and stilbenes (El-Seedi et al., 2012). Hydroxycinnamic acids are considered potent antitumor agents due to the presence of α, β-unsaturation, acting as Michael acceptors (De et al., 2011). Relevant hydroxycinnamic acids include caffeic, ferulic, p-coumaric, and sinapic acids, and the most representative hydroxybenzoic acids include gallic, vanillic, syringic, protocatechuic, and p-hydroxybenzoic acids. Phenolic acids can be found in vegetable-derived foods including cereals, legumes, soybeans, coffee, tea, rosemary, thyme, apples, various berries, plums, cherries, and citrus fruits (Clifford and Scalbert, 2000; El-Seedi et al., 2012). Different health effects have been related to phenolic acids. Chlorogenic acid has exhibited its anticancer potential by inducing differentiation through an increase of KHSRP, p53, and p21, a decrease of poor differentiation-related genes *c-Myc* and *CD44*, and downregulation of oncogenic miRNA-17 family members in cancer cell lines (Huang et al., 2019). Other mechanisms involve epigenetic regulation; gallic acid inhibits DNMT1 activity through the negative regulation of p-Akt, reducing the nuclear import and stability of DNMT1. Potential epigenetic targets include *CCNE2*, *CCND3*, *CDKN1A*, and *CCNB1* genes, which play important roles in the GADD45 signaling pathway (Weng et al., 2018).

#### Flavonoids

Flavonoids are ubiquitous compounds in plants that are responsible for the fragrance, color, and flavor of fruits, seeds, and flowers, with important roles in pollination and protecting plants from ultraviolet (UV) light and acting as detoxifying agents and as signaling molecules, and they may play important roles in cold and heat acclimation (Koes et al., 1994; Panche et al., 2016). There are nearly 6,000 flavonoid-related compounds, including their derivatives flavanones, flavones, isoflavones, flavanols, flavonols, and anthocyanidins. Benzo-γ-pyrone is the basic chemical structure of flavonoids characterized by the presence of 15 carbon atoms as the base skeleton, organized in the form C6–C3–C6 (A+C–B) (two benzenic rings A and B) and linked by a unit of three carbons that may or not form a third-ring structure (pyran ring C). Flavonoids can occur as aglycones and as hydroxylated, methylated, and glycosylated derivatives and have great relevance for the sensory quality of citrus fruits. For example, flavonoids such as naringerin and neohesperidin are responsible for bitterness (Wang et al., 2017). Regular consumers of tea may have intakes of over 1,000 mg/day; however, normal diets only provide between 20 and 200 mg/day (Birt and Jeffery, 2013), and a regular dietary intake of flavonoids (500 mg/day) has been related to a diminished mortality risk (Bondonno et al., 2019).

##### Flavanones

The chemical structure is based on two benzene rings, A–B (the flavan core), bound by a dihydropyrone ring C, chirality at C3 of the C ring, and the absence of double-bound at the C2–C3 position, with 100 glycosides and 350 aglycones as known members (Barreca et al., 2017). The principal flavanones comprise naringenin, hesperidin, eriodictyol, taxifolin, didymin, and eriocitrin, regularly found in citric fruits and juices such as oranges, mandarins, and lemon (Khan et al., 2014; Barreca et al., 2017). The beneficial health effects related to the consumption of citric fruits have been linked to flavanones such as naringenin through modulation of the PI3K/Akt pathway and the nuclear translocation of the Nrf2 transcription factor, promoting the expression of HO-1 (heme oxygenase-1) and improving antioxidant defense (Zhang et al., 2017).

##### Flavones

Chemical characteristics of these flavonoids include a double bond between C3 and C4, a keto group at C4, and no substitution in C3. Flavones have a characteristic yellow color or can be colorless; they act as primary pigments in white flowers, as copigments in combination with anthocyanidins in blue flowers, and as plant-signaling molecules. Relevant flavones include apigenin, diosmin, chrysin, tangeretin, luteolin, 7,8-dihydroxyflavone, and 6-hydroxiflavone. Flavones are found in plants employed for preparing infusions such as chamomile and parsley. Apigenin glycosides are abundant in traditional teas (black, green, and oolong), while luteolin glycosides are found in rooibos tea (Hostetler et al., 2017; Seleem et al., 2017). Important bioactivities have been related to flavones; apigenin has demonstrated health benefits including the inhibition of cell proliferation, apoptosis induction, the prevention of stem-cell migration through the upregulation of p21 and p27, and the downregulation of NF-κB and PI3K/Akt pathways (Erdogan et al., 2016). Luteolin inhibits MCF-7 cell proliferation and cell-cycle arrest and activates apoptosis through the regulation of IGF-1-dependent IGF-1R and p-Akt without disruption of ERK1/2 phosphorylation (Sabzichi et al., 2014).

##### Isoflavones

Isoflavones differ from flavones because of the phenyl group located in C3 instead of in C2 in the pyran ring, and some of their derivatives can form a D ring (e.g., rotenoid) (Marais et al., 2006). Isoflavones represent the most abundant flavonoids in soybeans, in soy-derived products (tofu, soymilk, soybean flour) (Jung et al., 2000; Preedy, 2013; Terahara, 2015), and in green and mung beans. In humans, isoflavones may act as phytoestrogens because of their similarity to 17-β-estradiol (Křížová et al., 2019). Isoflavones may be found as conjugated forms with acetyl, malonyl glycosides (e.g., genistin, daidzin, and glycitin), or aglycones (e.g., genistein, daidzein, and glycitein) (Zaheer and Humayoun Akhtar, 2017). Isoflavones may regulate cancer-related signaling pathways. Genistein and daidzein treatment of ovarian cancer cells inhibits invasion and cell migration in a dose-dependent manner through the downregulation of FAK and the PI3K/Akt/GSK signaling pathway and modulates p21 and cyclin D1 expression, related to the presence of ERβ (Chan et al., 2018).

##### Flavonols

Constituted of a 3-hydroxyflavone backbone, flavonols entertain an unsaturation between C2 and C3, an OH^−^ at C3, and a carbonyl group at C4, and along with flavones and anthocyanidins, they act as copigments to strengthen the color of flowers (Bueno et al., 2012). Flavonols are usually found as β-*O*-glycoside conjugates to facilitate storage in vacuoles (glucose being the most common conjugate) (Aherne and O’Brien, 2002). Flavonol-rich dietary sources include fresh capers, dried parsley, elderberry juice, rocket lettuce, red onions, fresh cranberries, fresh figs, apples, red wine, and tea (Di Matteo et al., 2007; Kozłowska and Szostak-Wegierek, 2014; Haytowitz et al., 2018). The principal flavonols include kaempferol, quercetin, fisetin, isorhamnetin, and myricetin, and their consumption has been related to a broad spectrum of health benefits. Different mechanisms are involved in the anticancer effects of flavonoids. Quercetin-3-*O*-glucoside inhibits cell growth, arrests the cell cycle in phase S, induces apoptosis through caspase-3 activation, and inhibits topoisomerase II activity in human hepatic-cancer cells (Sudan and Rupasinghe, 2014). Other mechanisms include apoptosis induction through modification of the BAX/Bcl-2 ratio and evoking paclitaxel chemosensitization by the downregulation of MDR-1 (associated with paclitaxel resistance) in myricetin-treated ovarian cells (Zheng et al., 2017).

##### Flavanols

Flavanols (also known as flavan-3-ols or catechins) have a pyran ring with an OH^−^ at C3, the B ring is bound to C2, and there is a lack of a double bond between C2 and C3 (allowing for two chiral centers). Flavan-3-ols are found either in free form or as gallic acid esters in different food sources such as apples, black tea, green tea, dark chocolate, and red wine (Rothwell et al., 2013). Relevant flavanols include the following: (+)-catechin; (+)-gallocatechin; (−)-epicatechin; (−)-epigallocatechin; (−)-epicatechin 3-gallate; (−)-epigallocatechin 3-gallate; theaflavin; theaflavin 3-gallate; theaflavin 3′-gallate; theaflavin 3,3′-digallate; and thearubigins (Haytowitz et al., 2018). Several health benefits have been related to flavanols. Lung cancer cells treated with (−)-epigallocatechin 3-gallate decreased the cell migration induced by human neutrophil elastase and induced α-1 antitrypsin through PI3K-pathway regulation (Xiaokaiti et al., 2015).

##### Proanthocyanidins

Proanthocyanidins (condensed tannins) are linked by C–C (sometimes by C–O–C) bonds, varying in the degree of polymerization (Rue et al., 2018). According to interflavan linkages, proanthocyanidins are classified as type A or type B. Type A lacks interflavan linkage but possesses another bond between the OH- from A ring and the C2 of C ring (C2–O–C7 or C2–O–C5) and type B with bonds between the C4 of B ring and either C6 or C8 of C ring (C4–C6 or C4–C8) (Rauf et al., 2019). Proanthocyanidins, which are composed of catechin or epicatechin subunits, are known as procyanidins; if they are composed of epigallocatechin subunits, they are called prodelphinidins. Proanthocyanidins confer astringency and bitterness and are regularly found in natural sources such as the fruits/seeds/peels of *Vitus vinifera*, *Punica granatum*, and *Theobroma cacao*, the leaves of *Fructus crataegi* and *Eucalyptus* spp., the flowers of *Rosa rugosa* and *Nymphaea tetragona*, and the roots/stems of *Rheum palmatum* and *Ipomoea batatas* (Yang et al., 2018). Procyanidins along with flavones possess high antioxidant activity (Lv et al., 2015); catechin-related compounds are the most powerful flavonoids against reactive oxygen species (ROS), with a broad spectrum of health benefits. Grape proanthocyanidins have been associated with a decrease of UVB-induced photocarcinogenesis in SKH-1 mice through the regulation of immunosuppression by decreasing the expression of IL-10 and increasing that of IL-12 (Katiyar et al., 2017). Proanthocyanidins also inhibit cell proliferation by means of the modulation of miRNA expression (Wang et al., 2019).

##### Anthocyanidins

Anthocyanidins are composed of ring A linked to ring C, which is bound in C3 to ring B, with no carbonyl group in C4 and two unsaturations in ring B at the O–C2 and C3–C4 positions. Anthocyanidins are salt derivatives from the flavylium cation with a positive charge in the oxygen atom. Their color is pH-dependent, with red predominating under acidic conditions, whereas blue predominates under alkaline conditions (Laleh et al., 2005). Anthocyanidins can be found as aglycones, but when they are conjugated into a glycoside, they are known as anthocyanins (Khoo et al., 2017). Anthocyanidins act as naturally occurring pigments found in the flowers and fruits of many plants that confer red, pink, blue, or violet shades (Kumar and Pandey, 2013) and that occur in the outer cell layer of many edible products including blueberries, strawberries, raspberries, red wine, and red onion. The most representative anthocyanidins are cyanidin, delphinidin, pelargonidin, peonidin, malvidin, and petunidin (Castañeda-Ovando et al., 2009; Rothwell et al., 2013; Haytowitz et al., 2018). Many anthocyanidin-rich plants have been employed in traditional folk medicine and their effects have been extensively studied. A phase 0 clinical trial showed that an anthocyanin-rich raspberry lozenge administered to patients with oral squamous cell carcinomas (OSCC) for 14 days caused a reduction in the expression of prosurvival genes *AURKA*, *BIRC5,* and *EGFR*, and downregulation of proinflammatory genes *NFKB1*, *PTGS1*, and *PTGS2* (Knobloch et al., 2016).

#### Stilbenoids

Stilbenoids are nonflavonoid polyphenols derived from the phenylpropanoid pathway, in the form of hydroxylated derivatives of stilbene backbone C6–C2–C6 (two aromatic rings linked by a methylene bridge), with two possible planar configurations (*cis* or *trans*). Stilbenoids are usually found as aglycones, glycosidic/methoxyl conjugates, or oligomeric units (viniferins). Stilbenoids act as phytoalexins and 1,000 of these compounds have been identified to date (Xiao et al., 2008; Mekinić et al., 2016). Stilbenoid-rich sources include the plants of the Gnetaceae, Pinaceae, Cyperaceae, Fabaceae, and Dipterocarpaceae families; however, their content is <10% of that found in the Vitaceae family, and the richest sources of stilbenoids are wine, berries, and grape juice (Niesen et al., 2013; El Khawand et al., 2018). Resveratrol represents by far the most important compounds of its kind, followed by gnetol, piceid, astringin, pterostilbene, piceatannol, viniferins, etc. The bioactivities of stilbenoids include anticancer effects. Pterostilbene has shown upregulation of PTEN in prostate cancer cells and xenografts through the reduction of levels of oncogenic miR-17, miR-20a, and miR-106b (Dhar et al., 2015), thus highlighting the potential health effects of stilbenoids in terms of their being promising candidates as novel therapeutic agents.

### Absorption and Metabolism of Polyphenols

There are several considerations for the development of new drugs, including bioaccessibility and bioavailability. Bioaccessibility is the fraction released from the food matrix into the intestinal milieu, rendering the drug bioavailable (Dima et al., 2020), whereas bioavailability is the extent of the drug absorbed that reaches the systemic circulation, with the drug becoming available at the site of action (Chow, 2014).

Ingested polyphenols are subjected to biotransformation in the GI tract by either digestive enzymes or the gut microbiota and may impact their bioactivities. The majority of polyphenols are released in the stomach (65%) and small intestine (10%) (Bouayed et al., 2011). The main sites of polyphenol absorption include the intestine and the colon (5–10% of the ingested polyphenols), whereas unabsorbed polyphenolics accumulate at mM concentrations in the large intestine, where the gut microbiota will exert biotransformation (Cardona et al., 2013) because complex polyphenols cannot be absorbed without modifications (Deprez et al., 2001). The gut microbiota involved in the biotransformation of polyphenols includes *Eubacterium* spp., *Clostridium* spp., *Bifidobacterium* spp., and *Lactobacillus* spp. (Marín et al., 2015). Biotransformed polyphenols are absorbed through the intestinal wall, transported to the liver where hepatic enzymes will break down (phase I metabolism) or conjugate (phase II metabolism) polyphenolics, and then they are distributed to target organs or eliminated in urine. The biotransformation of polyphenols may limit biological effects, and this may explain the discrepancy between *in vitro* and *in vivo* effects. For example, although many metabolites of anthocyanins can be found in urine, parent compounds are not detectable, possibly due to full metabolization (Agulló et al., 2020).

Another example is curcumin, which has low bioavailability and poor absorption, and the majority of the ingested curcumin is detected in the form of phase II metabolism-derived products, whereas the parent compound is scarcely detectable in the organism (Liu et al., 2016; Tsuda, 2018). The gut microbiota plays a significant role in the metabolism of curcumin, especially *Escherichia coli*, which converts curcumin into tetrahydrocurcumin (Hassaninasab et al., 2011). Biotransformation is not always linked to the loss of bioactivity; the oxidative metabolites of curcumin possess important biological effects (Edwards et al., 2017). However, like the majority of polyphenols, after passing through the GI, 90% of curcumin is excreted (Metzler et al., 2013); this is significant in that 10% of the ingested curcumin is responsible for its biological effects. The low bioavailability and complex metabolism of polyphenols render it difficult to present recommendations concerning their daily intake. The high variability of results of *in vivo* experiments and clinical trials is attributed to the poor absorption and metabolism of polyphenols; however, their safety and the ease of obtaining make them ideal candidates for the treatment of many diseases.

### Anticancer Activities of Polyphenols Against the Foremost Malignant Tumors

Cancer constitutes an important public health concern worldwide with 19.3 million new cases and 10 million deaths in 2020. Principal cancer types include lung, colorectal, stomach, liver, breast, esophagus, prostate, and cervix uteri (Sung et al., 2021). Cancer development is closely related to unhealthy nutritional habits; the low consumption of fruits and vegetables (<800 g/day) has been related to an increase of 30–50% in the incidence of colorectal cancer (Vargas and Thompson, 2012; Aune et al., 2017). Plant-derived compounds are widely utilized by individuals due to their cost accessibility, the belief in better effectiveness compared to medical prescription drugs, and the trend toward the use of products of natural origin. The contribution of plant-derived natural compounds to the pharmaceutical field is extensive; important examples include aescin, morphine, paclitaxel, and vincristine and, in the most recent two decades, more than 30% of US Food and Drug Administration (FDA)-approved drugs are derived from natural compounds (Li F. et al., 2019).

Polyphenols may act as antioxidants through two main mechanisms as follows: first, phenolic groups accept an electron to form relatively stable phenoxyl radicals, preventing oxidative damage in cellular components. Second, OH^−^ groups act as hydrogen donors and interact directly with reactive nitrogen species (RNS) and ROS (Leopoldini et al., 2011), which could explain their preventive role in oxidative damage.

Polyphenols provide protection from cancer risk factors, including tobacco, alcohol, unhealthy diets, sedentarism, and even those related to carcinogenic infections caused by pathogens such as the hepatitis B/C virus (HBV; HCV), the Epstein-Barr virus, and the human papillomavirus (HPV).

Nicotine represents the most toxic factor of tobacco, may lead to excessive cell proliferation through an increase in oxidative stress, and also has been related to an improvement of the invasiveness of lung and breast cancer cells (Bose et al., 2005; Dasgupta et al., 2009). Resveratrol prevents nicotine-induced cell proliferation through the MAPK signaling pathway by means of the downregulation of p-ERK in pancreatic cells (Chowdhury et al., 2018a). Alcohol consumption has been related to the development of colorectal cancer (Nishihara et al., 2014). Epigallocatechin 3-gallate (EGCG) inhibits ERK and activates JNK, thus fostering apoptotic cell death by the release of cytochrome c in human colon cancer cells (Cerezo-Guisado et al., 2015). Healthy food habits improve the health status of persons. The kaempferol present in apples and onions suppresses the expression of MMP-9 (related to metastasis progression) *via* the inactivation of the MAPK/AP-1 pathway in breast cancer cells (Li et al., 2015). HCV promotes the proteasomal degradation of pRb through the E6 ubiquitin-dependent mechanism, thus interfering with cell-cycle regulation and the response to cellular DNA damage. Treatment with theaflavins prevents the entry of HCV into hepatocytes but does not prevent viral replication (Chowdhury et al., 2018b); however, it represents a promising preventive approach for future malignancy induced by HCV infection. High-risk HPV represents other infectious agents of relevance for the development of malignant tumors; they account for approximately 25% of cases of HNSCC (HPV-16), and virtually all cervical cancers are caused by high-risk HPV (16 and 18). The combination of TriCurin polyphenols (curcumin, epicatechin 3-gallate, and resveratrol) reduces mRNA and the protein levels of E6 and E7, leading to the accumulation of p53 and pRb, thus decreasing tumor weight and cell proliferation by 86.3 and 19.9%, respectively (Piao et al., 2017). The effects of TriCurin on HNSCC appear promising, considering that this cancer is the sixth most prevalent malignancy worldwide (Shield et al., 2017).

Despite the fact that the bioactivities of polyphenols can often be limited by bioavailability, the detoxification metabolism, and the individual variability index, their wide range of health benefits is not limited to a single type of cancer or to a single mechanism of action. Therefore, polyphenols represent promising therapeutic agents for different cancers.

## Activities of Polyphenols in Relevant Cancer-Driving Signaling Pathways

### p53 Tumor Suppressor

#### p53 Overview

p53 represents the most important human tumor suppressor and a central element for cell-cycle control and apoptosis. p53 is composed of 393 amino acid (aa) residues and includes the following six domains: two N-terminus transactivation domains (TAD, including TAD1 and TAD2); a proline-rich domain (PRD); a central DNA-binding domain (DBD); a tetramerization domain (TD); and a C-terminus regulatory domain (CRD) (a rich-lysine region). The acidic nature of TAD (∼20%) contributes to the efficacy of the transactivation (Raj and Attardi, 2017). TAD are important for interaction with regulators including Mdm2 and MdmX and for the recruitment of chromatin modifiers CBP/p300, the latter prompting chromatin opening and p53 stabilization through the acetylation of CRD, preventing its ubiquitination (Raj and Attardi, 2017). The DBD contains six “hotspots” where the most frequent mutations occur in cancer. While R248Q and R273H disrupt p53/DNA binding, others produce local (R248Q; R273H) and global (R175H; R282W) conformational distortions (Brosh and Rotter, 2009; Baugh et al., 2018). TD facilitates p53 tetramerization, contains a nuclear export signal hidden in a tetrameric form that allows for nuclear accumulation, also influences the strength and conformation of DNA/p53 complexes, and is important for protein-protein interactions (CK2, PKC, and RelA bind to p53 through TD) (Chène, 2001; Gencel-Augusto and Lozano, 2020). CRD is required for the binding of promoters and structural changes in DBD (Laptenko et al., 2015) and undergoes extensive posttranslational modifications (PTM) on Lys residues.

p53 is under strict regulation because of its role as a central hub in the signal transduction of many cellular processes. In fact, while p53-null mice can live, those lacking Mdm2 and those that are incapable of regulating p53 die (Jones et al., 1995). The p53 half-life accounts for from 5 to 20 min in nearly all cell types, but after stress signals, senescence, or DNA damage, its stability is increased (Giaccia and Kastan, 1998). Negative regulators of p53 include Mdm2 and MdmX. Mdm2 promotes Lys ubiquitination at the C-terminus, targeting p53 for proteasomal degradation and abolishing the acetylation essential for the p53-mediated stress response (Tang et al., 2008). MdmX regulates p53 by direct interaction with TAD independent of E3-ubiquitin ligase activity (Raj and Attardi, 2017); however, MdmX can associate with Mdm2, enhancing its E3-ligase activity (Badciong and Haas, 2002). Recently, it has been demonstrated that MdmX inhibits the p53/DNA-binding function in association with CK1α (Wei et al., 2016). All of these control mechanisms highlight the importance of p53 regulation, which renders it an important therapeutic target due to its central role in cell fate control.

#### Polyphenols and Their Regulation on p53

**FIGURE 2:**
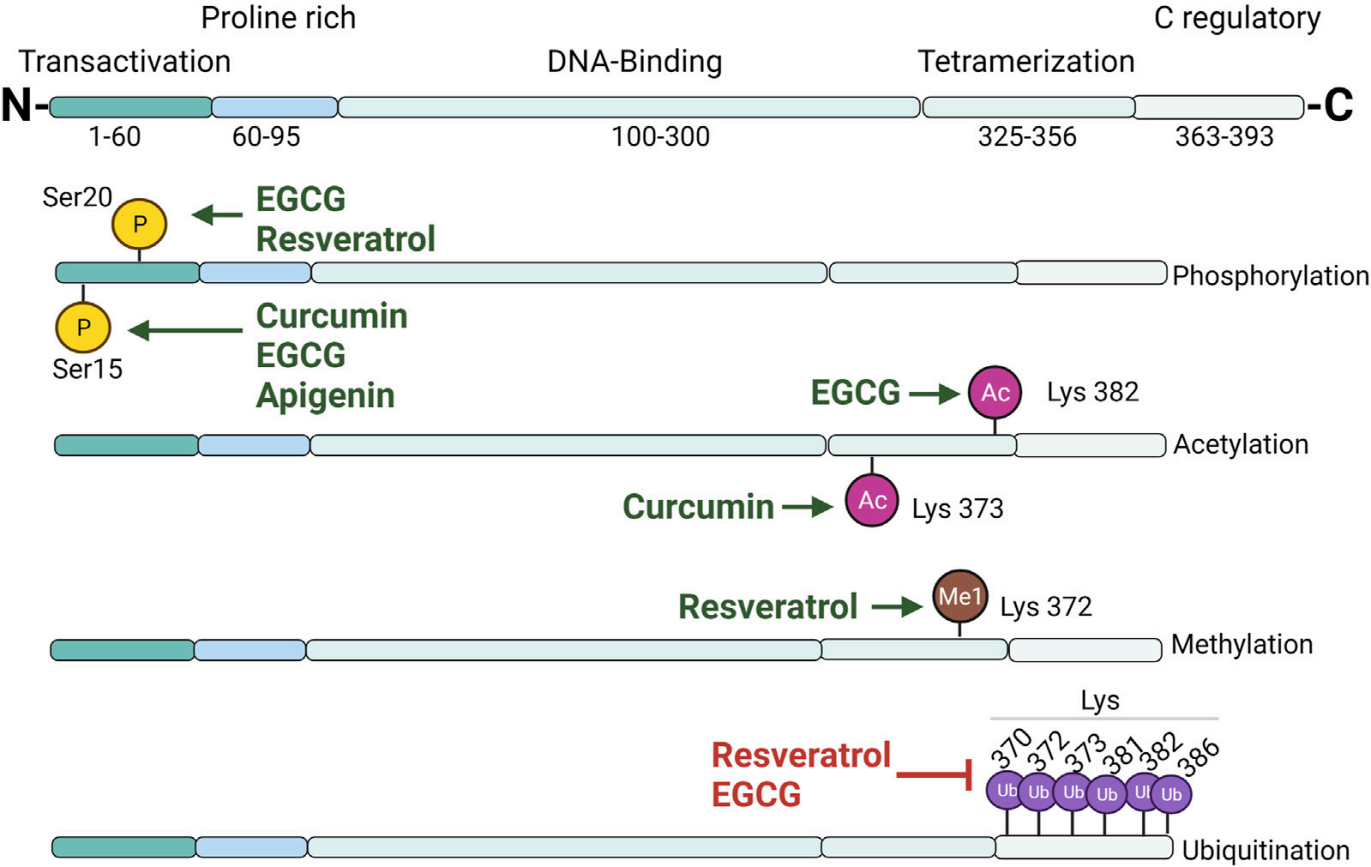
Overview of p53 domain structure and posttranslational modifications induced by polyphenols. Principal sites where polyphenols induce p53 posttranslational modifications (phosphorylation, acetylation, methylation, or ubiquitination) are plotted. TAD, transactivation domain; PRD, proline-rich domain; DBD, DNA-binding domain; TD, tetramerization domain; CRD, C-terminal regulatory domain.

p53 control is carried out by a variety of mechanisms, and the regulatory activities of polyphenols on p53 are widely reported in the literature. p53 undergoes extensive PTM (Figure 2), including phosphorylation, acetylation, ubiquitination, and methylation, which influence its stability, localization, and function; in addition, polyphenols may influence the posttranslational status of p53. It was recently described that curcumin promotes hyperphosphorylation in Ser15, thus promoting the expression of proapoptotic *Bex* genes in neuroblastoma cells (Sidhar and Giri, 2017). However, curcumin may also impair p53 folding into the required conformation for its phosphorylation, which affects its tumor-suppression function (Moos et al., 2004). Curcumin may alter p53/p300 interaction through p53 acetylation (Lys373), leading to the transcription of *BAX*, *PUMA*, and *Noxa*, thus enabling p53-mediated apoptosis in breast cancer cells (Sen et al., 2011). p53/p300 Interaction is important, considering that the genotoxic stress-related transcriptional activity of p53 is regulated by its interaction with its transcriptional coactivator p300. Nrf2 plays a protective role against oxidative stress in mammals by the regulation of antioxidant and detoxifying enzyme transcription (Saha et al., 2020). Dalton’s lymphoma has low levels of Nrf2; treatment with curcumin restores Nrf2 messenger RNA (mRNA) levels and enhances the binding of the protein Nrf2 to ARE and the NF-2E consensus sequence, thus increasing the levels of endogenous antioxidants and enhancing the general antioxidant status. Interestingly, curcumin increased p53 mRNA and protein levels, and this increase was related to the stabilization of Nrf2 expression (Das and Vinayak, 2015). Nrf2 induces the expression of the antioxidant enzyme NQO1 that, aside from its primary function, forms a complex with p53, leading to its stabilization in curcumin-treated cervical cancer cells (Patiño-Morales et al., 2020).

Several anticancer effects have been linked to treatment with resveratrol. Resveratrol induces phosphorylation in Ser20, promoting p53 stabilization, thus leading to the activation of target genes and the induction of apoptosis (Hernandez-Valencia et al., 2018). Polyphenols may contribute to p53 stabilization through the prevention of Mdm2-mediated ubiquitination or by the modulation of deubiquitinating enzymes. Ubiquitination plays an important role in p53 degradation and localization. USP10 (a cytosolic deubiquitinating enzyme) with an affinity for p53 reverses Mdm2-mediated ubiquitination, cytoplasmatic degradation, and nuclear export and impacts the transcriptional activity of p53 (Sun and Dai, 2014). Resveratrol binds to G3BP1 and interrupts the G3BP1/USP10 interaction, releasing USP10 and promoting its deubiquitinating activity, increasing p53-mediated apoptosis in melanoma (Oi et al., 2015). Without the disruption of the p53/USP10 complex by G3BP1, p53-deubiquitination results were affected, leading to its proteasomal degradation.

SET7/9 methyltransferases regulate p53 through monomethylation in Lys372, resulting in protein stabilization and activation. Resveratrol treatment in colon cancer cells induces p53 methylation, leading to *BAX* and *PUMA* gene expression. The absence of SET7/9 abolishes p53 proapoptotic effects; hence, their presence appears to be essential for cell death (Liu L. D. et al., 2019).

Flavonols possess important regulatory activities on p53. Quercetin upregulates p53 mRNA and protein levels as well as increases caspase 3/7 activity in mesothelioma (Lee et al., 2015). Combined treatments of curcumin and quercetin augment phosphorylation and acetylation levels in lung carcinogenesis with the downregulation of Bcl2 and the upregulation of p21 and *BAX*, leading to apoptosis (Zhang and Zhang, 2018). An important premise with respect to polyphenols relies on their apparent affinity for inducing regulatory effects in cancer cells, but not in normal cells. Quercetin induces cytotoxic effects in leukemic and breast cancer cells but did not affect normal cells through direct quercetin/DNA interaction, thus increasing p53 and p-p53 levels, leading to the induction of apoptosis and cell-cycle arrest in the S phase. Quercetin reduced tumors, improved lifespan, and had no adverse effects in mice (Srivastava et al., 2016). A combination of quercetin with the chemotherapeutic MG132 (a specific 26S proteasome inhibitor) appears promising. This combination demonstrated a synergistic effect, extending the half-life of p53 from 74 to 184 min, stabilizing p53 through Ser15 phosphorylation, and preventing ubiquitination in HepG2 cells (Tanigawa et al., 2008).

Kaempferol, which is another important flavonol, also exhibits relevant bioactivities in p53 in cancer. Kaempferol treatment of human cancer cell lines containing mutant p53 led to apoptotic cell death with an increase of cleaved PARP and caspase (3, 7, 9) levels, the release of cytochrome c, and DNA fragmentation (Lee et al., 2014). In another study, human colon cancer cell lines (HCT116, HCT15, and SW480) were treated with kaempferol; molecular markers cleaved PARP and caspase-3 increased after treatment. The proapoptotic effects of kaempferol may be exerted through the regulation of different pathways; the expression of p53, p21, and p-p38 was upregulated, whereas p-JNK and p-ERK were attenuated. Interestingly, the proapoptotic effects of kaempferol were related to an increase in the intracellular ROS level (Choi et al., 2018).

Catechins, present in many tea-derived products, also possess important regulatory activities. EGCG promotes p53 accumulation, increases transcriptional activity through phosphorylation on Ser15 and Ser20, and prevents p53/Mdm2 interaction, increasing the half-life from 40 to 90 min in lung cancer (Jin et al., 2013). EGCG increases p53 acetylation in Lys382, enhancing its stabilization and DNA binding, increases p21 expression, downregulates HDAC-4, -5, and -6, and stimulates apoptotic induction in lung cancer cells (Oya et al., 2017). Relevant mechanisms of EGCG regulation on p53 include direct interaction between p53 and p53. EGCG binds to the N-terminal domain of p53 (aa involved in this interaction include W23 and W25, F54, G52, and T55) and shields p53 TAD, which is the Mdm2 interaction site (involving p53 residues F19, L22, T23, L26, G58, E68, V75, and C77), thus inhibiting Mdm2-mediated ubiquitination (Nagata et al., 2014; Karakostis et al., 2016; Zhao et al., 2021).

Flavones have also been linked to bioactivities against important types of cancer. Apigenin modulates the balance between prosurvival and proapoptotic pathways by the activation of p53, the repression of STAT-3, and decreased ROS levels in lymphoma cells (Granato et al., 2017). Apigenin enhances the response to Cisplatin-induced apoptosis by disruption of the p53/Mdm2 interaction and favors MAPK-mediated p53 Ser15 phosphorylation, protecting it from proteasomal degradation (Liu et al., 2017). The combination of apigenin with TRAIL has been related to apoptotic effects on non-small-cell lung cancer (NSCLC) in a p53-dependent manner. This combination revealed a synergistic effect by increasing the mRNA levels of DR4, DR5, and protein p53. TRAIL interaction with DR4/5 leads to the formation of the death-inducing signaling complex (DISC), with the subsequent binding of caspase-8, which activates the caspase cascade. The use of the p53 inhibitor (PFT-α) abolished the effect of the combined treatment; hence, these effects showed to be p53-dependent. Proapoptotic effects on lung cancer cells were related to the upregulation of BAX and Bad and to a prominent reduction of Bcl-2 and Bcl-xL levels (Chen et al., 2016).

As discussed so far, polyphenols appear to possess promising activities in p53 regulation through different mechanisms; however, several studies must be performed to elucidate the fully implicated mechanisms and consequences of polyphenol treatments in p53 regulation for the development of new, efficient, and safe cancer therapies.

### MAPK Pathway

#### MAPK Overview

MAPK belong to serine/threonine kinases central to one of the principal signaling cascades involved in the control of cell growth, differentiation, survival, and cell death. MAPK signaling is activated in response to intra- and extracellular signals; these signals activate transmembrane glycoproteins of the tyrosine kinase receptor type, leading to the regulation of target genes. MAPK signaling cascades are composed of three main players as follows: the stress-activated protein kinase c-Jun NH2-terminal kinase (JNK), the stress-activated protein kinase 2 (SAPK2, p38), and the extracellular signal-regulated protein kinases (ERK1/2, p44/p42). JNK and p38 are activated by cytokines, hypoxia, genotoxicity, and oxidative stress; ERK is activated by mitogens and cytokines, principally by means of the activation of RAS family members (Rodríguez-Berriguete et al., 2012).

The dysregulation of MAPK can lead to cell transformation; the RAS–Raf–MEK–ERK axis is altered in 40% of human cancers, principally in RAS (30%) (Santarpia et al., 2012). RAS represents a family of GTPases composed of 150 G-proteins (HRAS, KRAS, and NRAS) and represents the first actors in the MAPK/ERK phosphorylation cascade (Johnson and Chen, 2012). Activation of RAS will result in ERK phosphorylation and activation; therefore, ERK translocates to the nucleus and promotes the activation of transcription factors such as c-Fos and c-Jun (Eblen, 2018).

JNK and p38 are known as stress-activated protein kinases. JNK translocates from the cytosol to the nuclei and evokes c-Jun activation through Ser63 and Ser73 phosphorylation, changing the expression patterns of BAX and Bcl-2 (Zhou et al., 2015). The role of JNK in cancer development is found in its multiple targets that are implicated in many cell-regulation mechanisms, such as STAT1/3, c-Jun, c-Myc, FOXO4, Bcl-2, ATF2, Smad2/3, PPARγ1, and RXRα (Dou et al., 2019). Multiple JNK-activated targets render JNK an important objective for targeted cancer therapies.

The p38 MAPK family comprises four isoforms expressed by different genes. Isoform p38α is ubiquitously expressed in all tissues, whereas isoforms β, γ, and δ are tissue-specific. Isoforms of p38 MAPK engage in redundant activities; however, the absence of p38α is lethal (Gerits et al., 2007). As many as 200–300 substrates are phosphorylated by p38 MAPK, including kinases involved in gene regulation, such as MSK1/2 (implicated in the regulation of transcription factors NF-κB p65 and STAT1/3), cytoplasmatic substrates such as cyclin D1, CDK inhibitors, Bcl-2 family proteins, and nuclear substrates including p53 (Cuadrado and Nebreda, 2010). Several interesting activities have been related to p38α, including, but not limited to, the suppression of ERK and JNK signaling by neutralizing RAS transformation, leading to senescence and cell-cycle arrest (Wang et al., 2002; Hui et al., 2007) and to the neutralization of tumorigenesis in lung, breast, colon, and liver through ROS production in response to oncogene activation, leading to the induction of p38-dependent apoptosis (Dolado et al., 2007). However, its role in tumor neutralization is only exerted at early stages; once the tumor is established, p38 promotes tumor growth and metastasis (Igea and Nebreda, 2015; Vidula et al., 2017).

The vast number of pathways regulated by MAPK make them ideal candidates for targeted therapies, and polyphenols may emerge as a promising alternative for the regulation of key players of MAPK pathways.

#### Polyphenols and Their Regulation on MAPK

**FIGURE 3:**
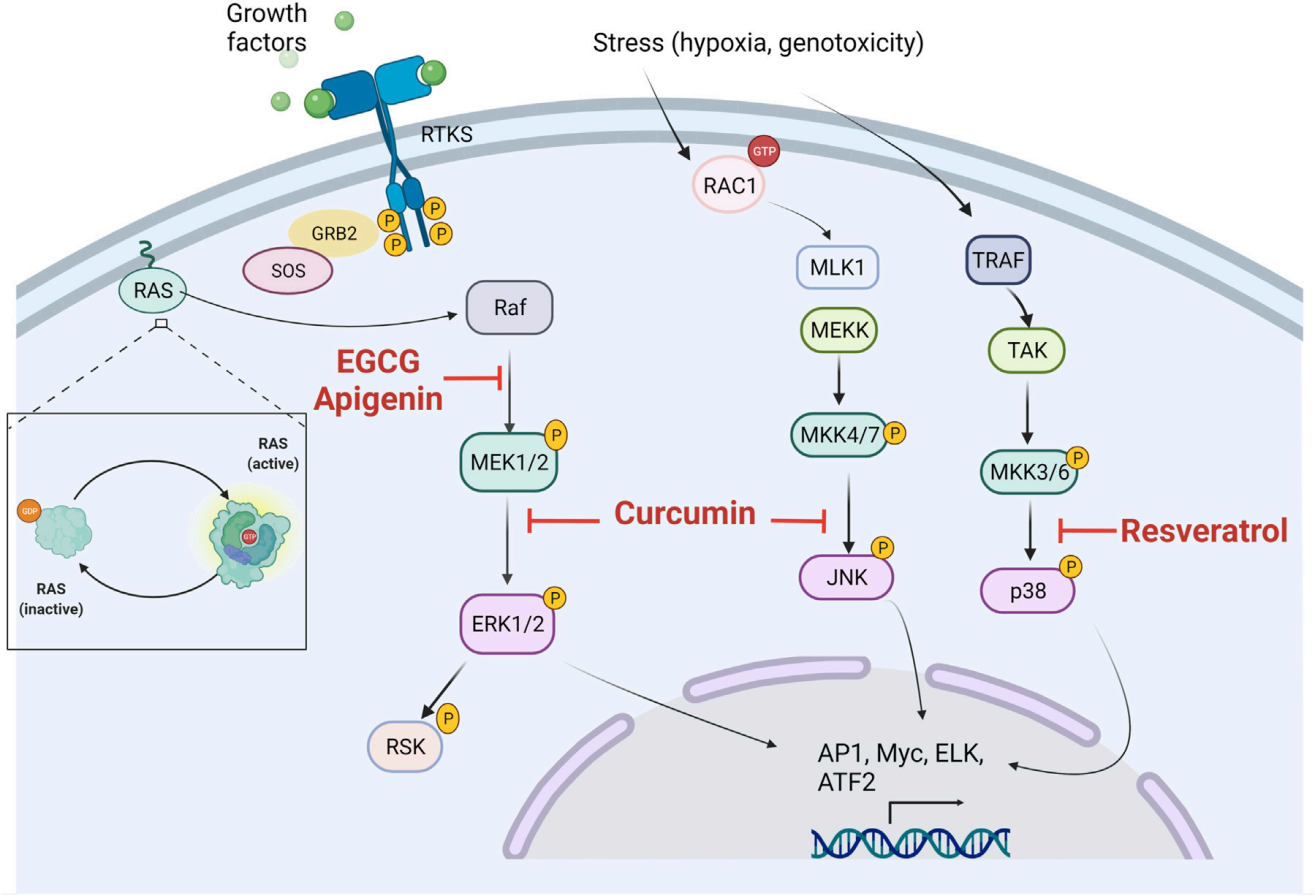
Potential sites of inhibitory actions of polyphenols in MAPK signaling pathways. ERK, extracellular signal-related kinases; JNK, c-Jun amino-terminal kinases; p38, p38 mitogen-activated protein kinase.

The bioactivities exerted by natural phenolic compounds rely on their different regulation mechanisms, which strongly contribute to their anticancer activities. Polyphenols have been associated with promising regulatory activities in MAPK pathways (Figure 3).

Curcumin as one of the most promising anticancer agents has exhibited different regulation mechanisms in MAPK. Curcumin treatment in Ishikawa cells (endometrial carcinoma) induces apoptosis, cell-cycle arrest in phase S, and the downregulation of ERK and Jun mRNA, as well as the reduction of the p-ERK-2/c-Jun pathway. Interestingly, curcumin reduced cell invasion by the downregulation of p-ERK/c-Jun and diminishing AP-1 synthesis, thus decreasing MMP2/9 transcription (Zhang et al., 2019). The effects of curcumin on MAPK pathways are not only limited to *in vitro* results. Curcumin treatment in the xenograft prostate cancer model demonstrated a reduction in tumor development. In this study, the mechanism involved in prostate cancer reduction by curcumin was related to a reduction of p-JNK, and curcumin was also effective for p-c-Jun reduction, leading to a decrease in Bcl-2 and Bcl-xL mRNA levels (Zhao W. et al., 2018). Stilbenoids have also revealed relevant activities for MAPK inhibition in different cancers. Resveratrol was effective in increasing p-p38 levels, leading to a decrease of Bcl-2 and an increase in Bad expression, as well as acting as a potent tumor growth inhibitor. The involvement of p38 was assessed using its inhibitor SB203580, which abolished the protective effects of the resveratrol treatment (Yuan et al., 2016). Urologic cancers represent a major concern. Resveratrol treatment reduces cell proliferation and the metastatic potential of renal-cancer cells. Resveratrol regulates ERK1/2 signaling pathways, specifically by altering the expression of ERK1/2, p-ERK1/2, E-cadherin, MMP-2, and MMP-9 (Zhao Y. et al., 2018). The apigenin treatment of melanoma-cancer cells A375 and C8161 promoted growth arrest through the downregulation of p-ERK1/2, p-Akt, and p-mTOR (Zhao et al., 2017). Tea polyphenols may act as potent anticancer agents alone or in combination with another chemotherapy. EGCG has demonstrated synergy with Sunitinib in cancer cell lines; both compounds decreased cell viability and suppressed the ERK pathway (Zhou et al., 2016). Kaempferol and quercetin stand as two of the most promising flavonols for cancer treatment. Quercetin possesses activities against colon cancer with mutant-type *KRAS* through JNK-pathway regulation; such activity results are very promising since *KRAS* is considered undruggable. In this study, quercetin selectively inhibited Akt and activated the p-JNK/c-Jun axis, leading to caspase-3 activation and subsequent apoptosis (Yang et al., 2019). In fact, flavonols can act as chemosensitizers. Kaempferol treatment overcomes resistance to 5-FU in resistant colon cancer cells. Concomitant treatment led to an increase in apoptosis, cell-cycle arrest, and modulated the protein expression of the JAK/STAT3, MAPK (ERK, p38), PI3K/Akt, and NF-κB involved in the progression and development of colorectal cancer (Riahi-Chebbi et al., 2019). All of these regulatory activities on MAPK pathways highlight the pharmacological importance that polyphenols may possess for the treatment of cancer by inhibiting these pathways; however, more research is necessary to fully elucidate the mechanisms involved.

### PI3K/Akt Pathway

#### PI3K/Akt/mTORC1 Pathway

The phosphatidylinositol 3-kinase (PI3K)/protein kinase B (Akt) and the mammalian target of rapamycin (mTOR) are signaling pathways that regulate survival and growth processes (Hemmings and Restuccia, 2012). These pathways are activated through several cellular stimuli and control essential cellular functions such as proliferation, transcription, translation, survival, and growth (Liu R. et al., 2020).

There are three classes of PI3K isoforms. Class I PI3K are heterodimer lipid kinases composed of the p110 catalytic subunit and the p85 regulatory subunit. Akt, also known as protein kinase B (due to its similarity with PKA and PKC) is a serine protein kinase, activated by growth factors in a PI3K-dependent manner (Hemmings and Restuccia, 2012). PI3K phosphorylates the inositol ring of the membrane phospholipid, phosphatidylinositol-4,5-biphosphate (PI-4,5-P2), to generate phosphatidylinositol-3,4,5-trisphosphate (PIP3) on the cytoplasmic side of the cellular membrane (Rathinaswamy and Burke, 2019). PIP3 recruits a subset of pleckstrin homology (PH) domain-containing proteins, such as the same protein kinase Akt and the constitutively active phosphoinositide-dependent kinase 1 (PDK1). In turn, PDK1 phosphorylates Akt into T308 (Ding et al., 2010); however, maximal activation of Akt requires its additional phosphorylation in S473 located at the carboxyl-terminus site, mediated by mTORC2 (Ikenoue et al., 2008).

mTOR is one of the downstream signaling targets of PI3K/Akt, which regulates several cellular processes, such as cell growth, motility, survival, and metabolism (Saxton and Sabatini, 2017). mTOR exists in two protein complexes, that is, mTORC1 and mTORC2, of which mTORC1 is directly inhibited by Rapamycin, a macrolide and antifungal compound; however, mTORC2 is insensitive to rapamycin (Saxton and Sabatini, 2017). mTORC1 controls cell growth and proliferation mainly by promoting transcription, translation, ribosome biogenesis, and autophagic regulation. On the other hand, mTORC2 regulates proliferation and survival primarily by phosphorylating several members of the AGC family of protein kinases (Fu and Hall, 2020).

Akt inhibits the tuberous sclerosis complex (TSC) that limits mTORC1 signaling. The TSC complex is composed of the following three subunits: TSC1 (Harmatin), TSC2 (Tuberin), and TBC1D7. Akt phosphorylates TSC2 in five residues (S939, S981, S1130, S1132, and T1462), leading to its inactivation. The TSC complex is a negative regulator of the small GTPase Rheb (RAS homolog enriched in brain) (Takahashi et al., 2003) *via* the stimulation of GTP hydrolysis. On the other hand, Rheb-GTP is translocated into the lysosomal membrane, where it directly interacts with the catalytic domain of mTOR, promoting its activation (Dibble and Manning, 2013; Kim and Guan, 2019).

PI3K/Akt/mTOR pathways are one of the main prosurvival pathways that are activated in human cancers (Noorolyai et al., 2019). The PI3K/Akt/mTOR pathway is found deregulated in cancer, which is characterized by an overexpression/hyperactivation of its effector proteins and alterations in the genes that encode those proteins (Revathidevi and Munirajan, 2019).

#### Polyphenols and PI3K/Akt/mTOR in Cancer

**FIGURE 4:**
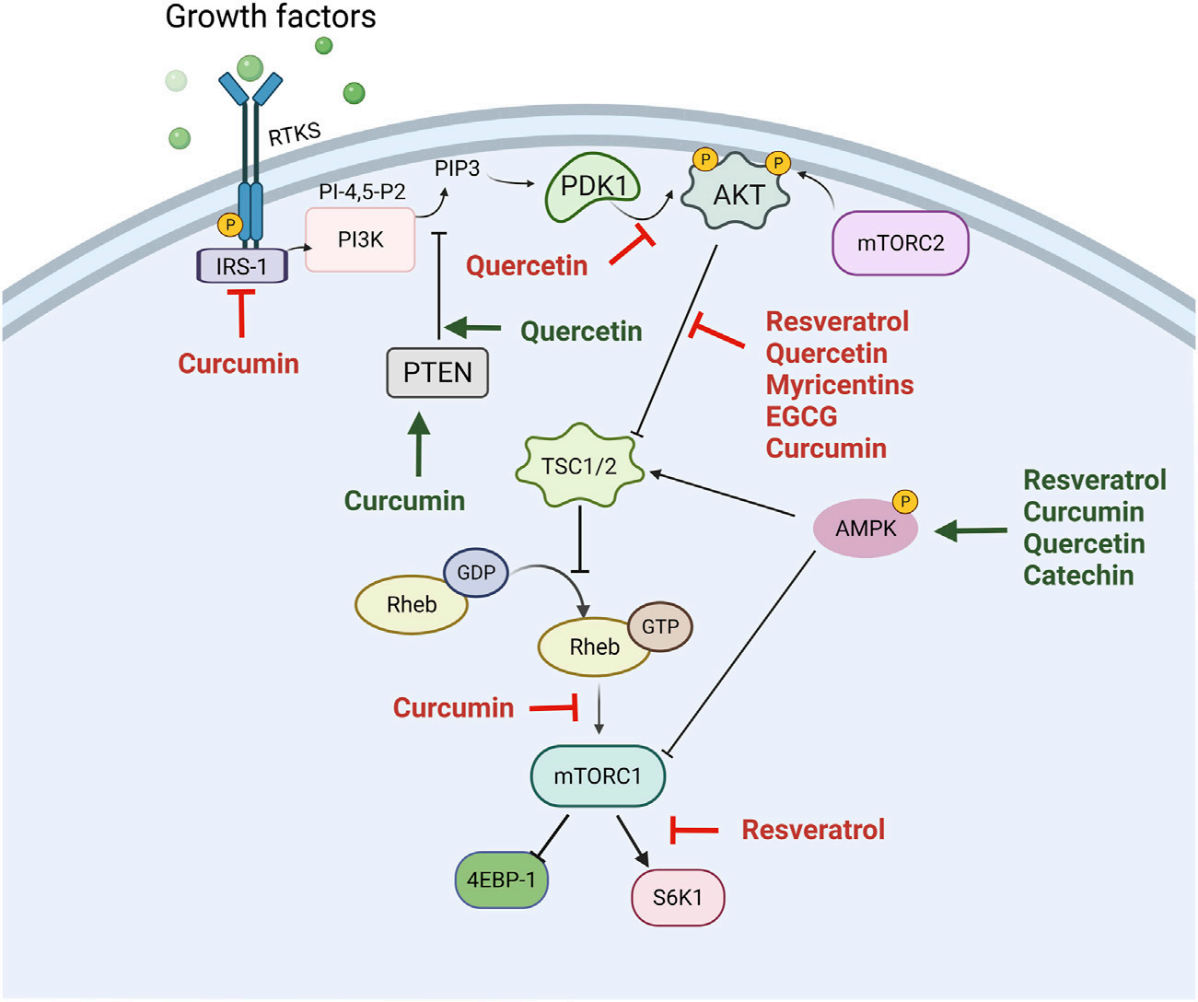
The inhibition of the PI3K/Akt/mTOR pathway by polyphenols in cancer. The phosphoinositide 3-kinase-AKT-mammalian target of rapamycin (PI3K/Akt/mTOR) pathway is hyperactivated in several cancers and is important in terms of tumor cell growth and survival. Activation of RTK, such as insulin-like growth factor-1 receptor (IGFR), by growth factors such as insulin, can initiate activation of intracellular pathways. Akt is phosphorylated downstream of PI3K with various effects, including the activation of mTOR. mTOR phosphorylates p70S6K and 4E-binding protein 1 (4EBP-1), which then leads to an increased translation of mRNA and cell growth. Polyphenols act by inhibiting this pathway by means of decreasing the phosphorylation of several protein kinases that are part of this signaling pathway. The green arrows indicate the activation of the pathway, while the red arrows indicate the inhibition of the pathway using different polyphenols.

The PI3K/Akt/mTOR pathway has been considered a major drug target due to its frequent hyperactivation in cancer (Liu Z. et al., 2020; Pevzner et al., 2021). Plant-derived natural compounds are one of the most reliable resources for cancer therapy. Several polyphenols, such as resveratrol, curcumin, apigenin, epigallocatechin 3-gallate, and quercetin, target numerous signaling pathways to exert tumor inhibitory and antiproliferative effects. One of these pathways is PI3K/Akt/mTOR (Figure 4).

Hyperactivation of Akt signaling is frequent in several cancers, which maintains a high oxidative state in a tumor microenvironment that is necessary for tumor adaptation. Antioxidants are proposed to exhibit anticancer roles by interfering with the tumor microenvironment. Resveratrol is a natural antioxidant and affects cellular oxidative stress and mitochondrial membrane potential by interfering with the PI3K/Akt signaling pathway in SCLC H446 cell lines (Li et al., 2020). In another study conducted by Jiao and collaborators (2015), resveratrol inhibited invasive behaviors *in vitro and in vivo* by PI3K/Akt/NF-κB suppression and the inhibition of MMP-2 secretion in glioblastoma (Jiao et al., 2015). An alternative antioxidant ubiquitously found in dietary sources with potential health-promising effects is quercetin, a bioactive flavonoid that has been identified as having antioxidant and anticancer effects. Many reports showed that quercetin possesses anticancer activities *via* Akt inhibition. Recently, it was found that quercetin attenuates cell survival, inflammation, and angiogenesis by modulating Akt signaling in lymphoma-bearing mice (Maurya and Vinayak, 2017).

The PI3K/Akt/mTOR is known to be involved in drug resistance (Liu R. et al., 2020). In agreement with this, resveratrol prevents resistance to Adriamycin by decreasing the expression of multidrug resistance protein (MRP1) through PI3K/Akt/Nrf2 in acute myeloid leukemia (Li Y. et al., 2019). Earlier researchers observed that green tea polyphenols such as EGCG act as a chemosensitizer, leading to minimizing chemoresistance and enhancing the chemosensitivity of tumor cells. EGCG and theaflavin (TF) synergistically inhibited the growth of HeLa cells through PI3K/Akt inhibition (Chakrabarty et al., 2019). TF or EGCG reduced the expression of both p85 (the regulatory subunit of PI3K) and phosphorylated Akt (Ser473), and interestingly, the reduction of protein expression was observed in a much higher amount in the case of the combination of these doses of polyphenols. This synergistic activity might be due to stronger microtubule depolymerization by the simultaneous binding of TF and EGCG to a different site on tubulin. This stronger microtubule depolymerization results in a higher G2/M arrest of the cell cycle and more drastic mitochondrial damage and synergistic augmentation of apoptosis (Chakrabarty et al., 2019).

Resveratrol possesses antitumor activity when used alone or in combination. Bian et al. examined that the coadministration of resveratrol and rapamycin significantly reduced the phosphorylation of Akt and p70S6K compared to treatment with rapamycin alone. This coadministration ablates mTOR function and prevents Akt activation, which overcomes the feedback activation of Akt and improves the antitumor effects (Bian et al., 2020). In an investigation, it was shown that combining the grape polyphenol resveratrol, quercetin, and catechin at equimolar concentrations inhibits mTOR signaling by means of a dual mechanism of PI3K/Akt and AMPK regulation and potentiates breast cancer to anti-EGFR therapy with Gefitinib, suggesting that this mixture may have synergistic effects against cancer (Castillo-Pichardo and Dharmawardhane, 2012).

PTEN is a tumor suppressor often deleted or mutated in a variety of cancers at a high frequency (Codrich et al., 2021; Zhang et al., 2021). It acts as a phosphatase that specifically catalyzes the dephosphorylation of the 3-phosphate of the inositol ring in phosphatidylinositol (3,4,5)-trisphosphate (PIP3), leading to the biphosphate product PIP2. The dephosphorylation of PIP3 results in the inactivation of the PI3K/Akt signaling pathway, because PIP3 is critical for the activation of Akt.

Resveratrol possesses anticancer activity through upregulating the bone morphogenetic protein 7 (BMP7) in order to inactivate the PI3K/Akt signaling pathway through partly suppressing the phosphorylation of PTEN in colorectal cancer. On the other hand, curcumin inhibits the proliferation of glioblastoma, and this effect is associated with the inhibition of both Akt and mTOR phosphorylation by promoting PTEN and p53 expression (Wang et al., 2020).

Accumulating evidence indicates that the PI3K/Akt/mTORC1 pathway is a negative regulator of autophagy (Yu et al., 2015). Autophagy is a process of the digestion of long-lived proteins and the damage of organelles and superfluously unwanted materials (Tavakol et al., 2019). According to the latter, it was demonstrated that resveratrol induces autophagic and apoptotic cell death through decreasing the phosphorylation of Akt in Ser473 and increasing the protein levels of phosphorylated AMPKα in Thr172. Resveratrol simultaneously enhanced the protein level of autophagy-associated proteins and the mRNA expression of the autophagic genes *Atg5, Atg12, Beclin-1,* and *LC3-II* in Cisplatin-resistant human oral cancer CAR cells (Chang et al., 2017). Interestingly, curcumin also induced autophagy and apoptosis in gastric cancer cells by activating p53 and the inhibition of the PI3K pathway (Fu et al., 2018).

Curcumin is a potent anticancer agent for the treatment of leukemia. A study by Zhou et al. in 2021 concluded that curcumin had stronger cytotoxic activity against acute myeloid leukemia cells compared with three other types of phytochemicals (epigallocatechin 3-gallate, genistein, and resveratrol). Mechanistically, curcumin treatment suppressed Akt activation, leading to cell-cycle arrest and apoptosis (Zhou et al., 2021).

mTORC1 functions as a downstream effector for PI3K/Akt, resulting in mTORC1 hyperactivation in a high percentage of human cancers (Saxton and Sabatini, 2017). Curcumin repressed mTORC1 signaling by two mechanisms involving the loss of IRS-1/Akt/PRAS40/Raptor/mTOR signaling and the activation of AMPK (Kaur and Moreau, 2021). These authors demonstrated that curcumin decreases the abundance of IRS-1 protein and inhibits the p-Akt (Ser473). Therefore, this led to a decrease in the phosphorylation of PRAS40 (Thr246), a negative regulator of mTORC1 (Kaur and Moreau, 2021).

On the other hand, in head-and-neck cancers, curcumin reduced the expression of phospholipase D1 (PLD1), the enzyme that catalyzes the production of phosphatidic acid (Borges et al., 2020). PLD1 binds to mTOR and displaces the mTOR-interacting protein (DEPTOR), an mTOR endogenous inhibitor, which results in mTORC1 activation and stabilization. In this manner, curcumin downregulates the PI3K/Akt/mTOR pathway and finally induces an arrest in the G2 phase of the cell cycle and induces cell death by apoptosis (Borges et al., 2020).

The PI3K/Akt pathway plays an important role in cancer progression, related to cell survival, growth, angiogenesis, and metastasis (Liu Z. et al., 2020; Lu et al., 2020). An important process in the progression of metastasis comprises vasculogenic mimicry (VM), the *de novo* formation of perfusable and vessel-like networks by aggressive tumor cells without endothelial cells. Several genes, such as vascular endothelial cadherin (*VE-cadherin*), participate in the formation of VM (Delgado-Bellido et al., 2017). Curcumin inhibits the VM of HCC cells by downregulating the Akt pathway (Chiablaem et al., 2014). Recently, it was shown that EGCG reduced p-Akt and Akt expression and reduced the ability of the VM of PC-3 cells. EGCG inhibited the nuclear localization of twist, followed by the downregulation of *VE-cadherin* expression, which in turn impaired the Akt pathway (Yeo et al., 2020). On the other hand, EGCG suppresses invasion and migration by preventing the cadherin switch and decreasing the expression level of TCF8/ZEB1, β-catenin, and vimentin in pancreatic cancer. Mechanistically, EGCG inhibited the Akt pathways in a time-dependent manner by suppressing IGFR phosphorylation and inducing Akt degradation (Wei et al., 2019). It has been reported that quercetin suppresses the mobility of breast cancer by the inhibition of glycolysis through the Akt-mTOR pathway and the activation of autophagy (Jia et al., 2018). Recently, it was shown that flavonoids quercetin and myricetin suppressed the HGF and TGF-α induced migration of HuH7 cells due to the attenuation of the PIK3/Akt pathway (Yamada et al., 2020).

### RAS Oncogene

#### RAS Overview

RAS proteins are eukaryotic small GTPases that cycle back and forth between the GDP-bound inactive state and the GTP-bound active state. RAS-GTP leads to the activation of various signaling pathways, such as MAPK, PI3K, and RAL-GEF, promoting a variety of crucial cellular processes including cell proliferation, differentiation, and survival in response to extracellular stimuli. RAS family members are encoded by three highly homologous genes that encode four highly homologous proteins: HRAS, NRAS, KRAS4A, and KRAS4B (the results of alternative splicing at the C-terminus) (Weiss, 2020).

RAS signaling responds to many extracellular stimuli, such as soluble growth factors. Growth factor binding to cell-surface receptors creates intracellular docking sites for adaptor molecules and signal-relay proteins that recruit and activate guanine nucleotide-exchange factors (GEF). GEF displace guanine nucleotides from RAS and permit passive biding to GTP, which is abundant in the cytosol. On the other hand, RAS proteins are negatively regulated by GTPase-activating proteins (GAP), which markedly stimulate intrinsic GTPase activity by stabilizing a high energy-transition state that occurs during the RAS-GTP hydrolysis reaction (Fernández-Medarde et al., 2021).

Human cancers frequently express mutant RAS proteins, termed “oncogenic RAS.” RAS oncogene mutations are those that result in a persistent GTP-bound, active state. The most common oncogenic RAS mutation comprises the substitution of a single amino acid at positions 12, 13, or 61, which induces a constitutively active RAS phenotype (Muñoz-Maldonado et al., 2019).

*KRAS* is the most frequently mutated RAS family member that can potentiate tumor-promoting activity. These *KRAS* alterations have been identified in 25% of all cancers, such as blood, breast, colorectal, gynecological, lung, prostate, and pancreatic cancer, in which some cancers, pancreatic cancer (90%), colorectal cancer (52%), and lung adenocarcinoma (32%) have extremely high mutation rates (Mustachio et al., 2021).

#### RAS Oncogene and Polyphenols

The RAS oncogene is particularly difficult to target with specific therapeutics. These RAS-mutated cancers respond poorly to standard chemotherapy; thus, targeted approaches need to be found (Sheffels and Kortum, 2021). Significant efficacy has been demonstrated in the treatment of tumors with various polyphenols, in particular the majority of polyphenols that entertain specificity toward tumor cells that express mutated *KRAS* and not so in normal cells.

Several flavonoids were tested on *HRAS-*transformed cells. Of these, apigenin, kaempferol, and genistein were able to reverse the transformed phenotypes, affecting cellular proliferation, morphological change, and colony formation in soft agar. The antitumor effect of resveratrol on oncogenic RAS was explored using a WR-21 cell line derived from a submandibular salivary adenocarcinoma. These WR-21 cells express an activated human *HRAS* transgene (mutated Asp12) RAS protein, as well as p53. This established that resveratrol inhibited cell proliferation and induced cell death by apoptosis, through p53 without direct modulation of the expression of both mRNA and the protein of mutant *HRAS* (Young et al., 2005).

Manna et al. examined the *in vivo* antitumor efficiency of black tea polyphenols such as theaflavin, EGCG, and ECG in lung cancer. Treatment with these polyphenols inhibited benzo(a)pyrene-induced lung carcinogenesis in mice; moreover, it significantly reduced the expression of proliferation-associated genes such as *HRAS*, *c-Myc*, and *cyclin D1* compared to the B(a)P-treated lung lesions (Manna et al., 2009).

It was shown that polyphenols such as curcumin and resveratrol, on being supplemented in a diet, can prevent the formation and growth of tumors by downregulating *KRAS* expression (Limtrakul et al., 2001; Saud et al., 2014). EGCG inhibited cell proliferation induced by oncogenic RAS in intestinal epithelial cells and blocked cell-cycle transition at the G1 phase *via* inhibition of *cyclin D1* expression, and EGCG exhibited a stronger inhibitory effect on cell proliferation in transformed cells than on nontransformed cells (Peng et al., 2006). The latter demonstrated the potential of the natural compound EGCG as effective adjuvant therapy for colon tumors bearing RAS mutations.

An *in vivo* investigation was conducted by Saud and collaborators (2014) to evaluate the preventive and antitumor effect of resveratrol using a genetically engineered mouse model for colorectal cancer that has a conditional knock-out of both copies of APC combined with a latent activated gain-of-function in the *KRASG12D* mutation specifically in the distal colon. The finding demonstrated that resveratrol orally administered at human equivalent doses (210 mg/day) prevented initial tumor formation and retarded the growth of established tumors. Resveratrol suppressed the expression of *KRAS* both *in vitro* and *in vivo* and induced the expression of miR-96, a microRNA (miRNA) previously shown to regulate *KRAS* translation. These data indicate that resveratrol can prevent the formation and growth of colorectal tumors by downregulating *KRAS* expression (Saud et al., 2014).

Oncogenic RAS has been shown to sensitize colon cancer cells to treatment with quercetin; moreover, this quercetin preferentially reduces the half-time life of the oncogenic RAS protein vs. the wild-type RAS (Psahoulia et al., 2007). Epicatechin-rich cocoa polyphenol extract inhibits the growth of human premalignant and malignant *KRAS*-activated pancreatic ductal adenocarcinoma. This finding demonstrated that both the extract and epicatechin alone reduced the GTP-bound active RAS protein level without having any effect on total protein. Moreover, they showed that this extract decreased PI3K/Akt and MAPK signaling by inhibiting *KRAS* activity (Siddique et al., 2012).

### Clinical Trials of Polyphenols With High Potential of Cancer Health Benefits

In recent times, polyphenols have gained importance as possible therapeutic agents, significantly increasing their use in clinical trials to explore potential health benefits in different cancers. Despite the abundance of studies in which curcumin, EGCG, resveratrol, quercetin, apigenin, and kaempferol have demonstrated excellent anticancer properties, the majority of these studies were performed in preclinical models. The bioactivities of polyphenols must also be investigated in humans because it cannot be assumed that the experimental results in cellular/animal models can be extrapolated to humans, principally due to differences in genetics and metabolism. The majority of these studies imply the exploration of pharmacokinetics, pharmacodynamics, safety, and the mechanisms by which these compounds reveal their effects. Currently, according to the US National Library of Medicine, 386,104 research studies can be consulted that have been conducted in all 50 US states and in 219 countries to date (August 2021), and 71, 39, 17, 14, 1, and 0 clinical trials related to different cancers using the polyphenols curcumin, EGCG, resveratrol, quercetin, apigenin, and kaempferol are available, with relevant studies listed in Table 1.

**TABLE 1:** Clinical trials of polyphenols-of-importance for cancer treatment.

Polyphenolic compound	Cancer type	Clinical trial Phase	*n*	Dose and trial length	Adverse and/or toxic effects	Effect	Endpoints	References
Curcumin	Precancerous lesions of the urinary bladder, Bowen’s disease, uterine cervical intraepithelial neoplasia, oral leukoplakia, and intestinal metaplasia	I	25	0.5–8.0 g/day; oral administration for 3 months	No	Improved histological status of 7 out of 25 patients with high risk or precancerous lesions at all applied doses	No dose-dependent effect was observed since histological studies showed improvement at nearly all doses	Chen et al. (2001)
							The recommended dose for a phase II clinical trial is 6–8 g/day	
	Multiple myeloma	I	15	3.0-4-0 g/day oral administration, combined with immune-modulatory drugs (IMD) or proteasome inhibitors (PI) for 1–3 months	Yes, two patients developed diarrhea	Decreased paraprotein and plasmacytosis levels by 38 and 59%, respectively	Curcumin, when used in a combination regimen in multiple myeloma patients, has comparable progression-free survival without the adverse effects of steroid-based combination therapies. Curcumin may be a viable alternative to corticosteroids in combination with an IMD or PI	Ramakrishna et al. (2020)
	Metastatic breast cancer	II	150	Paclitaxel 80 mg/m^2^ and intravenous curcumin 300 mg/week for 12 weeks	No, curcumin may decrease fatigue in the paclitaxel-treated patients	Tumor reduction by 50.7% in curcumin treatment compared with 33.3% placebo	Paclitaxel-curcumin combination therapy showed superiority over paclitaxel monotherapy in patients with advanced or metastatic breast cancer	Saghatelyan et al. (2020)
	Pancreatic cancer	II	25	8 g/day orally administered curcumin for over 1 year	No	Curcumin showed poor oral bioavailability. Two patients showed relevant clinical activity: one patient showed stable disease for more than 1.5 years; patient showed brief but consistent tumor regression (73%) accompanied by increased levels of serum cytokines (IL6, IL8, IL10, and IL1) in the range of 4-35-fold. Curcumin reduced the expression of NF-κB, COX-2, and pSTAT3 in mononuclear blood cells from patients	Oral curcumin is well tolerated and, despite its limited intestinal absorption, had a stable plasmatic level for 4 weeks, and had biological activity in some patients with pancreatic cancer	Dhillon et al. (2008)
	Pancreatic cancer	I/II	21	8 g/day of orally administered curcumin combined with Gemcitabine at 1,000 mg/m^2^ on days 1 and 8 plus 60 mg/m^2^ of S-1 every 3 weeks	Yes, two patients developed grade 1 diarrhea	Seventeen patients (81%) died during the study period. Median survival time of 161 days and 19% of 1-year survival rate. Patients reported an improvement in cancer or in chemotherapy-related symptoms (e.g., fatigue, pain, and constipation) after the initiation of curcumin intake. Among 18 evaluable patients, no patient experienced a partial or complete response, and five patients (28%) demonstrated stable disease according to RECIST	Curcumin treatment in combination with chemotherapy showed interesting outcomes concerning its tolerability and fewer side effects and even after 6 months of intake had no significant toxicity	Kanai et al. (2011)
	Metastatic colorectal cancer	IIa	28	2 g/days orally administered curcumin plus standard chemotherapy of folinic acid/5-Fluorouracil/Oxaliplatin (FOLFOX) every 2 weeks for 12 cycles	Yes, 10 events of low-grade diarrhea	Patients treated with curcumin plus FOLFOX had fewer negative changes in functional, symptom, and global health scores after trial than FOLFOX. Decreased plasma levels of CXCL1 by 1.7-fold for curcumin plus FOLFOX in comparison with FOLFOX alone	Curcumin resulted in safe and tolerable adjunct to FOLFOX chemotherapy in patients with metastatic colorectal cancer	Howells et al. (2019)
EGCG	Bladder cancer	II	31	800–1,200 mg/day of orally administered EGCG for 14–28 days prior to surgery	Yes, 4 patients developed headache with dose-dependent relation	Nearly, all tumor and normal tissue levels of ECG, EGC, and EC are zero; none of the catechins showed a trend toward differences in tissue. A dose-dependent correlation of catechins was found in plasma and urine. Possible chemoprotective activity by reduction of tumor biomarkers PCNA and clusterin	Demonstration of EGCG levels in plasma, urine, and bladder tissue and the establishment of a dose-response relationship, as did modulation of tissue biomarkers of proliferation and apoptosis. The pharmacodynamics and favorable bioactivity warrant further clinical studies of EGCG in bladder cancer prevention	Gee et al. (2017)
	Prostate cancer	II	97	200 mg/day of orally administered EGCG for 1 year	Yes, one patient developed nausea	Daily intake of EGCG, accumulated in plasma, and was well tolerated, and did not produce treatment-related adverse effects in men with ASAP or HGPIN. The intervention was not associated with increased risk of detection of high-grade disease in the group of men at end of the study. A decrease in PSA levels was related to EGCG treatment in patients	Data provided evidence of the safety of 200 mg EGCG, which showed a reduction of PSA levels. EGCG remains a possible candidate to be further tested for prostate cancer prevention or treatment	Kumar et al. (2015, 2016)
	Ovarian cancer	300	II	200 mg/day of Indole-3-Carbinol (I3C) and 200 mg/day of EGCG for 60 months with or without neoadjuvant chemotherapy	No	After 5 years of follow-up, maintenance therapy dramatically prolonged progression-free survival and overall survival compared to control	The endpoints were overall survival, progression-free survival, and the rate of patients with recurrent ovarian cancer with ascites after combined treatment within 5 years of follow-up	Kiselev et al. (2018)
						Median overall survival increased by 16 months in I3C and EGCG groups (from 44 to 60 months)		
						Median progression-free survival was 40 months in I3C and EGCG groups, while in groups without natural compounds treatment, the average progression-free survival was 23 months. The rate of patients with recurrent ovarian cancer with ascites after combined treatment was significantly less in maintenance therapy groups compared to the control		
	High-risk oral premalignant lesions	41	II	500–1,000 mg/m^2^ of orally administered TID EGCG for 12 weeks	Yes, some patients developed grade 1 or 2 insomnia, headache, nausea, and nervousness	The clinical response rate of patients with high-risk oral premalignant lesions (OPL) was higher in all EGCG groups compared to placebo, and such an effect seemed to be dose-dependent, but no statistical significance was found. EGCG treatment improved histology and was well tolerated. Higher mean baseline stromal VEGF correlated with clinical, but not with histologic, response. Other biomarkers (epithelial VEGF, p53, Ki-67, cyclin D1, and p16 promoter methylation) were not associated with a response or survival. p16 promoter methylation was associated with shorter cancer-free survival. Stromal VEGF and cyclin D1 expression were downregulated in clinically responsive EGCG-treated patients and upregulated in nonresponsive patients. EGCG may suppress OPL by reducing angiogenic stimulus (stromal VEGF). From the limited pharmacokinetic sampling available, there was no correlation between clinical or histologic response and levels of EGCG	Evaluation of the clinical and histologic response of high-risk OPL at 12 weeks with three different doses of EGCG. Qualitative and quantitative toxicities of EGCG, effects on the expression of biomarkers, and any correlation between treatment efficacy and/or toxicity with plasma concentrations of EGCG. Clinical and histological responses were assessed by complete or partial disappearance of lessons and by an increase of lesions	Tsao et al. (2009)
Resveratrol	Healthy volunteers	40	I	0.5–5.0 g/day orally administered for 29 days	Yes, 28 volunteers experienced mild nausea, flatulence, abdominal discomfort, and diarrhea	Resveratrol was safe and generally well tolerated by patients and showed mild gastrointestinal symptoms. Six metabolic conjugates in volunteers’ plasma were found, and the most abundant circulating metabolite was resveratrol-3-O-sulfate. Resveratrol decreased levels of IGF-1 and IGFBP-3, and there was a hint of pharmacodynamic activity in terms of the effect on circulating IGF protein levels at the 2.5-g dose, with a plasma level of 1.45 μM. IGF proteins may be used as potential biomarkers of pharmacological activity of resveratrol in humans because high levels of IGF have been related to several cancers; IGF is known to have antiapoptotic and mitogenic activities	Determination of clinical safety, pharmacokinetics, and effect on insulin-like growth factor axis at 29 days with four different doses of resveratrol. Safety was assessed by meeting the National Cancer Institute Common Terminology Criteria for Adverse Events (CTCAE). Plasma samples collected between days 21 and 28 of patients, parent compound, and metabolites of resveratrol were determined by HPLC. Pharmacodynamics was assessed in serum measuring IGF-1 and IGFBP-3 levels by ELISA	Brown et al. (2010)
	Colon cancer	8	I	Resveratrol was administered at 20 and 80 mg/day. Grape extract (GE) was administered at 0.073 and 0.114 mg/day. Both treatments were taken orally	No	No toxic effects were related to resveratrol or GE treatments. Patients treated with resveratrol/GE had no change in *Wnt* target gene expression in colon cancer. Resveratrol and GE seemed to have different effects on normal colonic mucosa, whereas inhibition of *Wnt* target gene expression was determined by microarray and confirmed by qRT-PCR of cyclinD1 and axinII. Resveratrol or GE may play a beneficial role in colon cancer prevention	The safety of resveratrol and GE was established by the report of any gastrointestinal symptoms. Clinical effects of resveratrol or GE were determined by the expression of Fz receptors, Wnt ligands, Wnt inhibitors, Wnt targets in normal mucosa, and colon cancer	Holcombe et al. (2009)
	Prostate cancer	14	I	500 up to 4,000 mg/day of Muscadine grape extract capsules MPX (containing 1.2 mg of ellagic acid, 9.2 μg of quercetin, and 4.4 μg of *trans*-resveratrol) administered orally for 28 days, with a follow-up of >2 years	Yes, four patients developed gastrointestinal symptoms, including grade 1 flatulence, soft stools, and eructation	Treatment was considered safe, and no tolerability issues were found in patients. At a 4,000 mg dose, ellagic acid, quercetin, and resveratrol were undetectable in the plasma. The lack of statistically significant within-patient change in PSADT and shortening in PSADT in 36% of the patients raise concerns about the efficacy of MPX. Median within-patient PSADT increased by 5.3 months (not significant). No patients experienced a maintained decline in serum PSA from baseline	Assessment of safety, tolerability, pharmacokinetic parameters, and efficient doses of resveratrol. Safety and tolerability were evaluated according to National Cancer Institute’s CTCAE. Pharmacokinetics was estimated by UPLC-ESI-MS/MS. Clinical benefits were determined by measuring PSA and PASDT	Paller et al. (2015)
	Breast cancer	39	I	50–50 mg BID of orally administered resveratrol for 3 months	Not reported	Total *trans*-resveratrol levels were detectable in 6/30 (20%) samples at baseline. A dose-response tendency was found with high-dose resveratrol resulted in higher levels at 4 and 12 weeks than after low-dose treatment. Total *cis*-resveratrol was not detectable in any (0/32) of the baseline serum samples. Major metabolites comprised glucuronides (93–100%), whereas sulfates comprised the remainder. The fraction of methylated RASSF-1α DNA decreased, whereas that of APC increased for 3 of 4 women after high-dose resveratrol compared to before treatment. The change in RASSF-1α methylation was directly related to the change in PGE2	Quantification of resveratrol and related metabolites in plasma of breast cancer patients. Establishment of resveratrol activities on methylation patterns of p16, RASSF-1α, APC, and CCND2. Modulation of PGE2 was evaluated	Zhu et al. (2012)
Quercetin	Different cancers (large bowel, ovarian, pancreas, melanoma, stomach, hepatoma, non-small-cell lung cancer, and renal)	51	I	60–1,700 mg/m^2^ every 3 weeks administered i.v.	No. However, patients had pain after injection of quercetin that lasted a few minutes. Three patients who received the dose of 1,700 mg/m^2^ developed renal toxicity	Quercetin plasma levels achieved immediately after injection were in the range of 200–400 µM at a 945-mg/m^2^ dose, with serum levels above 1 µM sustained up to 4 h. In 9 of 11 patients, lymphocyte protein tyrosine phosphorylation was inhibited following administration of quercetin at 1 h, which persisted to 16 h. Promising effects were observed in two patients. One patient with ovarian cancer refractory to Cisplatin (dose of 420 mg/m^2^) had a reduction of the CA 125 from 295 to 55 units/ml. Another patient with hepatoma had a reduction in α-fetoprotein from (460–40 units/ml). Quercetin showed to be safe for i.v. administration, and the reduction of tyrosine kinase could be a promising approach to antitumor activity	Determination of safety, pharmacokinetics by HPLC of patients’ plasma samples, and estimation of tyrosine kinase inhibition in lymphocytes isolated from patients by Western blot	Ferry et al. (1996)
	Blood malignancies; chemotherapy-derived oral mucositis	20	Pilot	250 mg capsule BID orally administered for 4 weeks	No	Patients received different chemotherapy regimens, but this was not related to the incidence of mucositis in the study population. The incidence of oral mucositis was lower in the quercetin group, but oral mucositis was more severe in the intervention group, which may be due to lower oral health status in the intervention group. No significant beneficial effect of quercetin was found in this study	Patients’ oral health was checked by an oral medicine specialist before chemotherapy was initiated. The World Health Organization (WHO) oral toxicity scale was used to evaluate oral mucositis. The endpoints were preventing incidence and onset of OM and the severity of oral mucositis	Kooshyar et al. (2017)

For quercetin, apigenin, and kaempferol, scarce evidence has been published to date in the literature on cancer clinical trials. An extensive search in the database clinicaltrials.gov resulted in only four completed studies of quercetin, and only two of these are reported in the literature. In the case of apigenin, the sole clinical trial (NCT00609310) has suspended status with no results reported, and for kaempferol, no cancer clinical trials in any phase have been reported to date. Results evidence the importance of continuing the carrying out of studies in different clinical phases, permitting the support of the great amount of preclinical evidence that has been found to date for the bioactivities of polyphenols, with the main objective of demonstrating both their safety and efficacy for the prevention and treatment of different types of cancer. Safety and tolerability are demonstrated in numerous studies, and some of the clinical trials listed in Table 1 include supporting evidence that curcumin, EGCG, resveratrol, and quercetin are safe for human clinical trials. Despite the problems related to the physicochemical properties of polyphenols, their administration route, pharmacokinetics, pharmacodynamics, and bioavailability, among others, involved factors that limit and impact their effectiveness and possible pharmacological action; these compounds may be considered serious candidates for cancer treatment. Because of the poor bioavailability of polyphenols and their extensive metabolism, high doses (up to a maximum of 12 g/day, depending on the type of compound tested) have been utilized by researchers in clinical trials. However, the results evidence the need for more research to increase the evidence and documentation of the bioactivities of these compounds in human subjects with well-controlled double-blind/placebo clinical trials for future therapeutic use, in order to establish their potential in terms of appropriate doses, the most effective routes of administration, in which types of cancer may they be most effective for treatment. Potential medicinal use, accessibility, low cost, safety, and toxicological profile, as well as multiple evidence from preclinical and clinical studies, make polyphenols important candidates for cancer treatment.

## Discussion

Polyphenols have gained attention as promising compounds with regulatory activities in several signaling pathways related to cancer development and progression. Understanding how polyphenols regulate cancer-associated mechanisms is important in the development of new therapies for cancer treatment. Polyphenols compose the third largest group of plant-derived chemical compounds after terpenes and alkaloids (Kennedy and Wightman, 2011), making them an important source of possible therapeutical agents given the great diversity of the compounds, from the simplest phenolic acids to polyphenols with a high degree of polymerization. The complexity of polyphenols will have an impact on their bioavailability and bioactivities. Oral administration is the most usual dosage form because it is safe, convenient for medication delivery, noninvasive, and painless, no sterile conditions are needed, both liquids and solids can be administered, it is cost-effective, and it can be self-administered. Nevertheless, oral drug delivery has disadvantages, including nonimmediate action (not suitable for emergency cases), patients must be conscious, absorption is variable among individuals, and some medications are not available in oral form because they are degraded in the GI tract and they may imply the transformation of the drug into a less active form or into toxic metabolites (Kerz et al., 2007; Vinarov et al., 2021). The absorption of polyphenols in GI differs according to their chemical nature. The main compounds to become absorbed are, in decreasing order, isoflavones, phenolic acids (caffeic and gallic), catechins, flavanones, and quercetin glucosides, whereas high-molecular-weight polyphenols, such as proanthocyanidins, catechins, and anthocyanins, are poorly absorbed (Manach et al., 2004). Functional groups may affect polyphenol absorption; glycosidic residues (the most common moieties) may render polyphenol absorption difficult in the small intestine or in the enzymatic activity of gut microbiota. However, this is not always true: some glycosylated metabolites of quercetin possess better bioavailability than aglycone itself (Velderrain-Rodríguez et al., 2014). All of these processes involved in polyphenol absorption may lead to changes in the molecular responses obtained *in vitro* and *in vivo* and must be considered in terms of their bioactivities; however, once absorbed and on their reaching target tissues, polyphenols may exert their bioactivities.

Nanotechnology has high importance in pharmaceutical formulations, targeted therapies, and high efficiency-controlled release. The use of nanotechnology may overcome the bioavailability issues of polyphenols and increase their bioactivity. The application of nanotechnology leads to an increase in the bioavailability and bioactivity of phytomedicine by reducing the size of the particles, by surface modification, and by entrapping the phytomedicine. Different types of compounds may be employed for nanoparticle formulations, including biopolymers, liposomes, quantum dots, polysaccharides, proteins, and metals. The efficiency of polyphenols may be enhanced by employing nanoparticles to reach specific tissues and diminish immunogenicity. The small sizes of nanoparticles (10–150 nm) ensure more efficient accumulation in tumors. Nanoparticles of <10 nm probably will be cleared by kidneys, whereas nanoparticles of >150 nm may be recognized and eliminated by macrophages (Kijanka et al., 2015). Nanoparticles significantly increase the efficiency of polyphenols against tumors. Curcumin-loaded nanoparticles have demonstrated better dose effectivity and bioactivity in cervical cancer cells (Zaman et al., 2016). Different nanoparticle-based therapies are approved by the FDA for the treatment of different cancers. As relevant examples, Myocet was approved in the year 2000 for the primary treatment of breast cancer, and VYXEOS was approved in August 2017 to treat acute myeloid leukemia. Therefore, the exploration of the use of polyphenol-loaded nanoparticles as novel anticancer therapies has a promising future.

Cancer represents a major public health concern around the globe, and despite the existence of a variety of therapies for its treatment, these therapies are often accompanied by adverse effects or toxicities in patients. Given the molecular complexity involved in cancer development and progression, novel treatments may be obtained using polyphenols. Polyphenolics are often recognized as safe products in several toxicity studies. According to reports of the European Food Safety Authority (EFSA), the daily recommended safe dose of curcumin is 0–3 mg/kg body weight (Kocaadam and Şanlier, 2017). The most promising polyphenols (curcumin, resveratrol, quercetin, and EGCG) and their use as possible therapeutic agents are being explored in clinical trials in different cancers including colon, breast, and prostate, to explore their clinical effects alone or in combination with chemotherapy.

**FIGURE 5:**
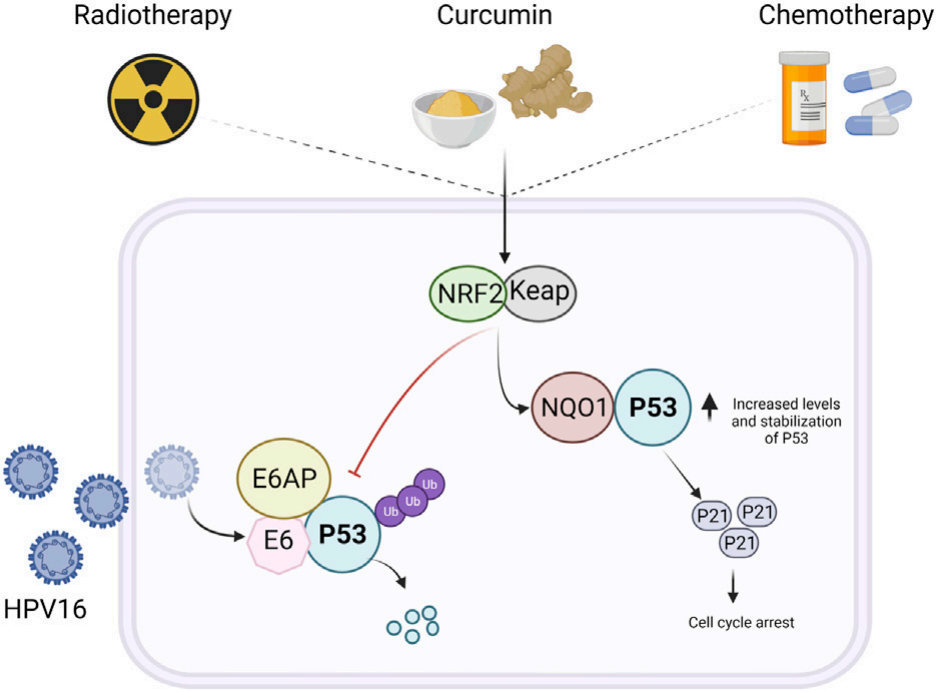
p53 Stabilization by curcumin and synergy with routine therapy for the treatment of cervical cancer. Curcumin activates the Kelch-like ECH-associated protein 1-nuclear factor (erythroid-derived 2)-like 2 (Keap1/Nrf2) pathway, leading to an increase in the levels of NAD(P)H:quinone oxidoreductase 1 (NQO1). NQO1 binds to p53, promoting the loss of the interaction between p53 and E6AP, a negative regulator, promoting p53 stabilization in cancer cells with wild-type p53, such as cervical cancer cells. Moreover, a promising area of study is that of evaluating the synergistic effect of curcumin with routine chemotherapy, thus procuring a better response to treatment and fewer adverse effects.

p53 plays a central role in many cellular processes, can be activated by diverse stimuli, and is followed by its corresponding response, including apoptosis, senescence, cell-cycle arrest, DNA repair, metabolism regulation, and differentiation (Aubrey et al., 2016). Although *p53* is the most studied gene of all time (Dolgin, 2017), many questions on its regulation in cells that will determine cellular fate remain unclear. Regulation of *p53* may be achieved by posttranslational mechanisms, mRNA-level modulation, protein stability, etc. Dietary polyphenols have important regulatory activities on *p53* (Figure 5), including protein stabilization by its interaction with proteins (NQO1) in cells treated with curcumin (Patiño-Morales et al., 2020); EGCG evokes the regulation of mRNA and protein levels (Chu et al., 2017) or the epigenetic regulation induced by resveratrol, leading to the reestablishment of *p53* (Chatterjee et al., 2019). These features of polyphenols highlight their importance and their possible therapeutic action against cancer; however, there are many questions concerning the specific cancer type for which they must be used or on their molecular mechanisms of action, which are still poorly understood, as well as the synergistic effect they may have with conventional chemotherapy (Figure 5). Therefore, further research is required to answer these questions in order to move forward to the pharmacological use of polyphenols for cancer.

The PI3K/Akt/mTOR pathway plays a major role in survival, growth, metastasis, and drug chemoresistance in cancer. This pathway comprises a major node that is frequently mutated or amplified in a wide variety of solid tumors (Manning and Cantley, 2007). Several novel anticancer agents targeting the PI3K/Akt/mTOR pathway have been developed for the treatment of various malignancies. For this reason, inhibiting any component of this pathway comprises a promising therapeutic strategy. Wortmannin and LY294002 were the first-generation PI3K inhibitors that belong to the nonisoform-specific category. However, Wortmannin with irreversible inhibition lacks selectivity and adverse effects resulted in the termination of its clinical trials (Mishra et al., 2021). LY294002 has poor solubility, bioavailability, and several adverse effects, such as fatigue, nausea, vomiting, diarrhea, and hyperglycemia (Esposito et al., 2019).

Dietary polyphenols, such as the resveratrol present in peanuts, catechins in green tea, curcumin in turmeric, and apigenin in onions, have been widely demonstrated that polyphenols have antitumor effects with selective cytotoxicity to cancer cells and few adverse effects to the patient. Several preclinical experimental studies have been developed to highlight the antitumor effect of resveratrol, curcumin, apigenin, EGCG, and quercetin on several tumors. In this review, we describe that these polyphenols can alter the function of multiple molecules effective in PI3K signaling, such as Akt, mTOR, PTEN, and PDK-1, through different mechanisms, avoiding cancer progression, drug resistance, angiogenesis, and metastasis.

Multiple *in vitro* and *in vivo* studies have shown that polyphenols decrease drug resistance. It was revealed that resveratrol decreases this drug resistance by reducing the expression of MRP1 and the efflux of Adriamycin in HL-60/ADR cells (Li F. et al., 2019) or by promoting cell death by autophagy. There is a catabolic process for bulk or selective encapsulated lysosomal degradation and the recycling of obsolete or damaged cytoplasmic cargo including proteins and organelles. Autophagy plays a dual role in cancer. On the one hand, the activation of autophagy in cancer cells promotes the efficacy of anticancer strategies, while on the other hand, it may promote cancer progression through the enhancement of cell survival (Tavakol et al., 2019). Resveratrol and curcumin induce autophagy and apoptosis through a decrease of the phosphorylation of Akt (S473) and AMPK or through *p53* activation (Chang et al., 2017). In contrast, in ovarian cancer, treatment with curcumin induces protective autophagy by inhibiting the Akt/mTOR pathway, resulting in resistance to chemotherapy. Interestingly, in these tumors, the inhibition of autophagy and curcumin therapy may provide a new perspective for clinical intervention (Liu Z. et al., 2019). However, more studies are needed to demonstrate the precise molecular mechanism and whether it is feasible to employ it in other types of cancer.

As mentioned previously, polyphenols inhibit the PI3K/Akt/mTOR pathway; however, these agents can interfere with other signaling cascades involved in cancer progression, such as MAPK and oncogenic RAS.

RAS proteins are small eukaryotic GTPases that cycle back and forth between the GDP-bound inactive state and the GTP-bound active state. The *KRAS* gene can simultaneously harbor multiple mutations that can potentiate tumor-promoting activity in several human cancers; thus, it is necessary to utilize a new therapeutic strategy to inhibit this oncoprotein and therefore the development of cancer (Mustachio et al., 2021). However, the *RAS* oncogene is particularly difficult to target with specific therapeutics. These *RAS*-mutated cancers respond poorly to standard chemotherapy; therefore, targeted approaches need to be found (Sheffels and Kortum, 2021).

Several studies both *in vitro* and *in vivo* have shown that polyphenols such as curcumin, resveratrol, and EGCG supplemented in a diet can prevent the formation and growth of tumors by downregulating *KRAS* expression (Limtrakul et al., 2001; Saud et al., 2014). EGCG inhibited cell proliferation induced by oncogenic RAS and exhibited a stronger inhibitory effect on cell proliferation in transformed cells than in nontransformed cells (Peng et al., 2006). Epicatechin reduced the GTP-bound active RAS protein level; moreover, it was demonstrated that this polyphenol decreased PI3K/Akt and MAPK signaling by inhibiting *KRAS* activity (Siddique et al., 2012). The ability of polyphenols to decrease RAS activity affords the possibility that polyphenols could be used for the targeting of many types of cancer that are caused by RAS activation, representing an attractive opportunity for the treatment of these tumors, which are characterized by being resistant to conventional chemotherapy. However, more studies are necessary to establish effectiveness either as monotherapy or as a combined therapy.

Despite the wide range of polyphenol health-related beneficial bioactivities in the regulation of cancer-related signaling pathways, we must consider the possible undesirable adverse effects caused by polyphenols. The similarity and ability of soy isoflavones to act as phytoestrogens may lead to undesired effects, principally in hormone-responsive diseases; Genistein treatment produces cell-cycle arrest and an improvement in mitochondrial functionality in T47D (low ERα/ERβ ratio), but not in MCF-7 (high ERα/ERβ ratio) and MDA-MB-231 (ER^−^) (Pons et al., 2014). Polyphenols are not exempt from toxicological adverse effects. Despite the fact that curcumin is recognized as safe, patients given oral doses of curcumin 10–12 g exhibited minor grade-1 toxic effects according to the World Health Organization (WHO) toxicological classification (Vareed et al., 2008). Although extensive evidence supports the antioxidant protective effects of curcumin, high concentrations may induce an increase in intracellular ROS production. It has been demonstrated that curcumin (2.5–5 µg/ml) induces mitochondrial and nuclear DNA damage, which could raise questions concerning our safety (Cao et al., 2006; Burgos-Morón et al., 2010). Polyphenols such as quercetin inhibit CYP 1A2, 2C9, 2C19, 3A4, and 2D6 (Rastogi and Jana, 2014); therefore, we must take care when drugs metabolized through these CYP are coadministered with quercetin. Despite these possible adverse effects, the majority of the evidence supports the beneficial health effects of polyphenols. However, it is important to continue the development of experimental studies and clinical trials that allow us to understand the mechanisms that are fully involved and the specificity for the different types of cancer that can be treated with polyphenols.

In conclusion, this review has provided an overview of the principal strengths of the most promising polyphenolic compounds for the regulation of important key players in cancer, which control a wide variety of cellular processes such as differentiation, proliferation, apoptosis, cell-cycle arrest, and the responses to inflammatory processes or oxidative stress. Therapies in current use for cancer are associated with several adverse effects that reduce the overall quality of life or may cause the death of patients. Polyphenols have been attracting attention due to their multiple bioactivities and could be an interesting alternative as therapeutic agents with the aim of being more effective and less toxic for cancer treatment. Bioavailability is an important parameter to be considered in the use of polyphenols as therapeutics in patients with cancer, due to the biotransformation processes that modify their structure and, possibly, bioactivities along their passage through gut and liver metabolism. The anticancer effects of polyphenols are known to modulate several signaling pathways including MAPK and PI3K/Akt, important tumor suppressors such as p53, and oncoproteins such as RAS isoforms. Several polyphenols including curcumin, resveratrol, quercetin, kaempferol, EGCG, and apigenin may upregulate the expression of the key players in these signaling pathways in several cancer types through a variety of distinct mechanisms of action. All of these considerations make polyphenols a promising source of therapeutics for cancer treatment; however, further research is needed to elucidate the complete mechanisms involved in the polyphenol-induced regulation of cancer.
